# Optimal Revascularization Strategy for Patients With ST-segment Elevation Myocardial Infarction and Multivessel Disease: A Pairwise and Network Meta-Analysis

**DOI:** 10.3389/fcvm.2021.695822

**Published:** 2022-01-05

**Authors:** Kongyong Cui, Dong Yin, Chenggang Zhu, Sheng Yuan, Shaoyu Wu, Lei Feng, Kefei Dou

**Affiliations:** ^1^Cardiometabolic Medicine Center, Fuwai Hospital, National Center for Cardiovascular Diseases, Chinese Academy of Medical Sciences and Peking Union Medical College, Beijing, China; ^2^Coronary Heart Disease Center, Fuwai Hospital, National Center for Cardiovascular Diseases, Chinese Academy of Medical Sciences and Peking Union Medical College, Beijing, China; ^3^State Key Laboratory of Cardiovascular Disease, Beijing, China; ^4^National Clinical Research Center for Cardiovascular Diseases, Beijing, China

**Keywords:** ST-segment elevation myocardial infarction, immediate complete revascularization, staged complete revascularization, culprit-only percutaneous coronary intervention, hard endpoints

## Abstract

**Background:** The relative benefit of immediate complete revascularization, staged complete revascularization, and culprit-only percutaneous coronary intervention (PCI) remains unclear in hemodynamically stable patients with ST-segment elevation myocardial infarction (STEMI) and multivessel disease. The aim of this study was to compare the clinical outcomes of the 3 PCI strategies in this population.

**Methods:** We followed a pre-specified protocol (PROSPERO number: CRD42020183801). A comprehensive search of the electronic databases including PubMed, EMBASE and Cochrane Library from inception through February 21, 2020 was conducted. Randomized trials evaluating the comparative efficacy and safety of at least 2 of the 3 PCI strategies were identified. The primary endpoint was the composite of cardiovascular mortality or myocardial infarction (MI) during the longest follow-up. Pairwise and network meta-analyses were performed with random-effects model.

**Results:** Eleven trials including 6,942 patients were analyzed. Pairwise meta-analysis noted that immediate complete revascularization and staged complete revascularization were respectively associated with a 52 and 27% reduction in the risk of cardiovascular death or MI (relative risk [RR] 0.48, 95% confidence interval [CI] 0.32–0.73, I^2^ = 0%; and RR 0.73, 95% CI 0.61–0.88, I^2^ = 0%, respectively), compared with culprit-only PCI. The risk of cardiovascular death or MI was not statistically different in staged and immediate complete revascularization groups (RR 0.88, 95% CI 0.45–1.72, I^2^ = 0%). Network meta-analysis obtained almost similar results compared with pairwise meta-analysis, and immediate complete revascularization had a 77% probability of being the best strategy for reducing cardiovascular death or MI among the 3 PCI strategies.

**Conclusion:** The current evidence suggests that both immediate and staged complete revascularization were associated with a reduction of cardiovascular death or MI compared with culprit-only PCI. Further trials are warranted to directly compare immediate vs. staged complete revascularization in this population.

**Systematic Review Registration:** https://www.crd.york.ac.uk/prospero/, PROSPERO [CRD42020183801].

## Introduction

In patients with ST-segment elevation myocardial infarction (STEMI) undergoing primary percutaneous coronary intervention (PCI), approximately 50% have one or more significant non-culprit vessels (1–3). Based on findings from observational studies (4) and earlier randomized controlled trials (RCTs) (5–7), previous guideline discouraged PCI of non-culprit lesions during primary PCI in hemodynamically stable patients, while routine staged PCI of non-culprit disease was not addressed unless there was spontaneous or inducible ischemia (8). However, the subsequent RCTs suggested significant benefit of immediate or staged complete revascularization compared with culprit-only PCI (9–12). Although most trials have reported significant reductions only in major adverse cardiovascular events, mainly driven by reduced incidences of repeat revascularization, meta-analyses of these trials demonstrated a significant benefit in terms of death or myocardial infarction (MI) in favor of the complete revascularization strategy (13, 14). The 2015 American College of Cardiology (ACC)/American Heart Association (AHA) and 2017 European Society of Cardiology (ESC) guidelines thus upgraded the recommendations for revascularization of non-culprit vessels during primary PCI or as a staged procedure (2, 3).

Nevertheless, the relative benefit of immediate complete revascularization, staged complete revascularization, and culprit-only PCI with regard to the hard endpoints in patients with STEMI and multivessel disease remains an unresolved issue. Although current guidelines give an equal level of recommendation to the two types of complete revascularization strategy (2, 3), non-culprit vessel PCI performed during primary PCI is different from that performed as a staged procedure from interventional and pathophysiological perspectives (15). Previous network meta-analyses showed that immediate complete revascularization might be the preferred strategy in this population due to the reduced risks of death or MI, death, MI and repeat revascularization. In contrast, staged complete revascularization did not significantly reduce death or MI compared with culprit-only PCI (16, 17). Encouragingly, however, the Complete vs. Culprit-Only Revascularization Strategies to Treat Multivessel Disease after Early PCI for STEMI (COMPLETE) trial showed that staged PCI of non-culprit vessels reduced the 3-year risk of cardiovascular death or MI than culprit-only PCI (18). In this setting, we performed the updated pairwise and network meta-analyses to compare the prognostic impact of the 3 PCI strategies for patients with STEMI and multivessel disease.

## Methods

### Search Strategy

We followed a pre-specified protocol (PROSPERO number: CRD42020183801). A comprehensive search of the electronic databases including PubMed, EMBASE and Cochrane Library from inception through February 21, 2020 was conducted by two independent investigators to identify pertinent articles published in English (SY and SW). Conference proceedings for the scientific sessions of the ACC, AHA, ESC, Transcatheter Cardiovascular Therapeutics and EuroPCR were also searched. The following medical subject headings and search terms were used: “myocardial infarction”, “multivessel”, “non-culprit”, “non-infarct”, “percutaneous coronary intervention”, “angioplasty”, “revascularization”, “random”, “randomly” and “randomized”. In addition, we examined the references of the identified articles, relevant reviews and meta-analyses to include other potentially eligible studies.

### Selection Criteria

Studies satisfying the following criteria were eligible: (1) hemodynamically stable patients with STEMI and multivessel disease; (2) RCTs evaluating the relative efficacy and safety of at least 2 of the 3 PCI strategies, i.e., immediate complete revascularization (PCI of culprit vessels as well as ≥1 non-culprit vessels during the index procedure), staged complete revascularization (PCI of culprit vessels during the index procedure, followed by separate PCI of non-culprit vessels before discharge or within a few weeks after STEMI), and culprit-only PCI (PCI of only culprit vessels, with subsequent revascularization warranted for ischemia); (3) trials with follow-up period of > 6 months; and (4) trials reported endpoint data of interest. Studies that allowed either an immediate or a staged complete revascularization in 1 randomization arm were categorized according to the predominant strategy (>75%). Of note, if neither of the two treatment strategies account for > 75% of the patients in a group, the study will be excluded. We also excluded studies only enrolling patients with chronic total occlusion (CTO) in non-culprit vessels or cardiogenic shock.

### Data Extraction and Quality Assessment

The following data was independently extracted by two authors through a standardized form for each study: first author, year of publication, study design, patient characteristics, quality indicators and clinical outcomes (LF and CZ). Differences in assessments were resolved by discussing with a third investigator (DY). The quality of RCTs were assessed by evaluating the following methodological criteria recommended by the Cochrane Collaboration (19). Trials with low risk of bias for at least 4 components were classified as having low risk of bias. Otherwise, they were considered as having high risk of bias.

### Endpoints

The primary efficacy endpoint was the composite of cardiovascular mortality or MI during the longest follow-up. All-cause mortality, cardiovascular mortality, MI and repeat revascularization were the secondary efficacy outcomes. Safety endpoints included contrast-associated acute kidney injury, stroke, major bleeding and stent thrombosis. All the endpoints were defined as reported in each study (Supplementary Table S1).

### Statistical Analysis

In pairwise meta-analysis, the relative risks (RRs) with 95% confidence intervals (CIs) were calculated using the Dersimonian and Lair random-effects model. Analysis was performed on intention-to-treat basis. Potential heterogeneity among studies was quantified with I^2^ statistic, and I^2^ > 50% was defined as statistical heterogeneity. For the primary endpoint, separate analyses were conducted according to the conditions described below: (1) trials with low risk of bias according to the Cochrane Collaboration's tool; (2) trials published in full text; (3) trials published after 2012; and (4) trials in that PCI of non-culprit vessels were mainly guided by angiography. Moreover, meta-regression analyses were carried out to assess the correlation of the following covariates with cardiovascular mortality or MI: age, gender, diabetes, hypertension, anterior MI, three-vessel disease, use of drug-eluting stent (DES) and duration of follow-up. To test whether the results were already definitive or may be implicitly influenced by novel data in the future, we conducted trial sequential analysis of the included RCTs for the primary endpoint, and the detailed methods were previously described (20, 21).

Network meta-analyses with a Bayesian framework were performed to combine both direct and indirect evidence about the 3 PCI strategies. Bayesian hierarchical random-effects models with directed acyclic graph models for general-purpose Markov chain Monte Carlo analysis were created with 20,000 tuning iterations and 50,000 simulation iterations. Convergence was assessed with the Brooks-Gelman-Rubin method and by visual inspection of convergence plots. Inconsistency was tested by contrasting direct with indirect evidence through node-splitting analysis. Of note, consistency model was utilized to draw conclusions, or an inconsistency model was adopted when statistical inconsistency was detected. The rank probability plot produced by the network meta-analysis was applied to present the best strategy. The risk of potential publication bias was assessed by visual inspection of funnel plots.

This study was performed according to the Preferred Reporting Items for Systematic Reviews and Meta-Analyses (PRISMA) statement (22). All *p* values were two-sided, and results were considered statistically significant at *P* < 0.05. Computations were performed using Review Manager 5.3 (Cochrane Center, Denmark), ADDIS (Aggregate Data Drug Information System, version 1.16.6) and the trial sequential analysis software version 0.9.5.10 Beta.

## Results

### Eligible Studies

**Figure 1:**
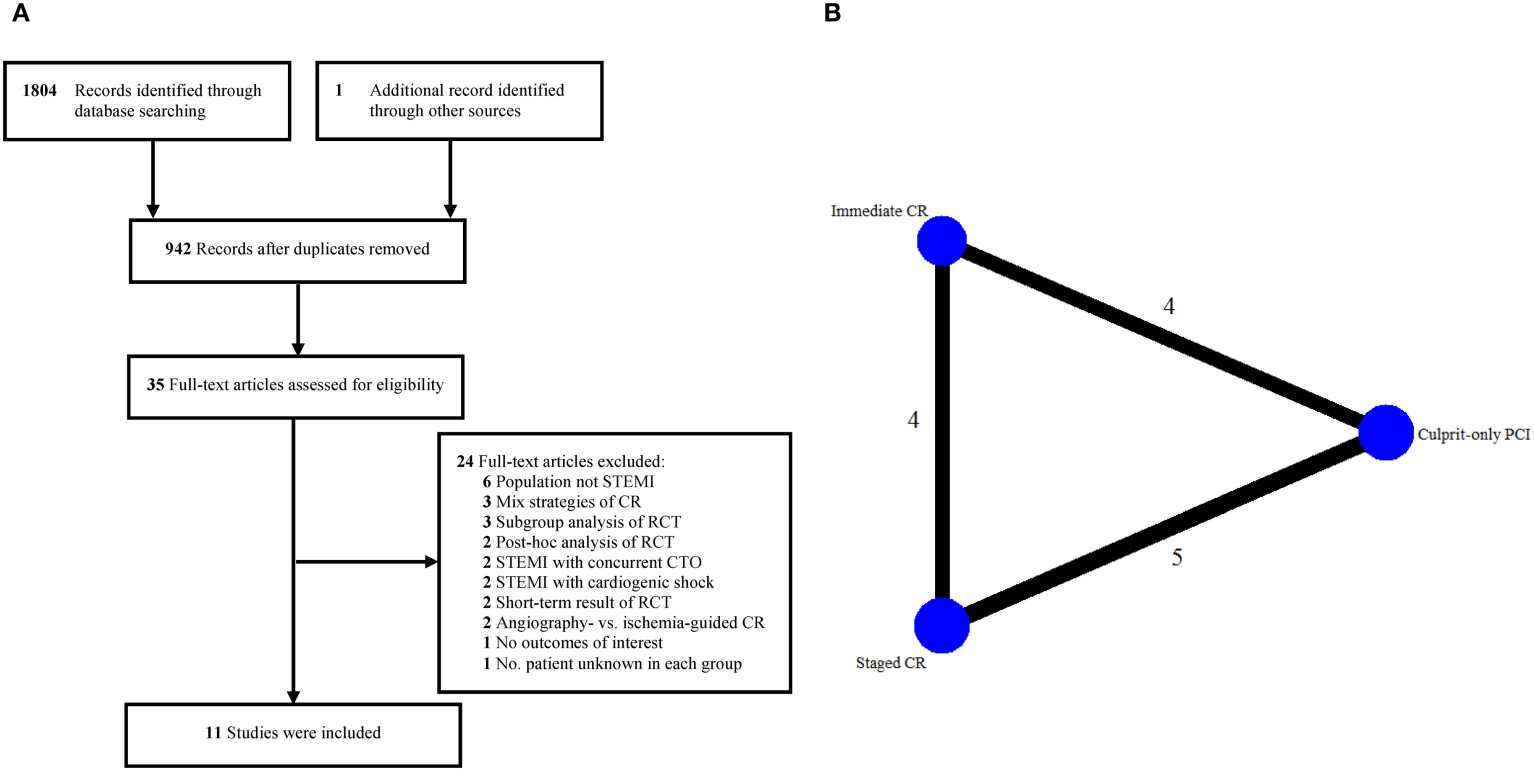
Eligible studies. **(A)** Flow diagram of included studies. **(B)** Network diagram. CR, complete revascularization; CTO, chronic total occlusion; PCI, percutaneous coronary intervention; RCT, randomized controlled trial; STEMI, ST-segment elevation myocardial infarction.

Eleven studies involving 6,942 patients were included in the final analysis (5–7, 9, 11, 12, 18, 23–26). The process of selecting studies and the network diagram are depicted in Figure 1. Notably, the Complete vs. Lesion-Only Primary PCI trial (CvLPRIT) and the study by Hamza et al. that involved both single-procedure and staged PCI of non-culprit vessels as part of the complete revascularization strategy were not included, considering that only 64 and 58% (<75%) of the patients underwent single-procedure multivessel PCI in the complete revascularization group, and they did not report separate data of the outcomes of interest (10, 27).

The main characteristics of the eligible studies are presented in Table 1. Three studies compared immediate complete revascularization vs. culprit-only PCI, 4 studies compared staged complete revascularization vs. culprit-only PCI, 3 studies compared staged vs. immediate complete revascularization, and 1 study compared all the 3 PCI strategies. Of note, the study conducted by Hlinomaz et al. were reported only in conference presentations (25). The identification of significant non-culprit stenosis requiring PCI was based on angiography in most of the studies, while fractional flow reserve (FFR) was mainly used in 3 studies (7, 11, 12). The timing of staged PCI was during hospitalization or within 57 days after primary PCI. The follow-up period ranged from 6 to 38 months. Overall, more than half of the included studies had a low risk of bias (Supplementary Figure S1) (6, 7, 9, 11, 12, 18).

**Table 1:** Main characteristics of the eligible studies.

**Study**	**No. patients**	**Interventions**	**Period**	**Region, center**	**Inclusion criteria**	**Exclusion criteria**	**Follow-up** **period, years**
Di Mario et al. (5)	69	Immediate CR vs. COR	NA	UK/Italy, multi	STEMI with multivessel disease; 1~3 lesions in non-IRA amenable to treated with stent	CS; LM disease; ≥1 CTOs; diffuse calcification or severe tortuosity; <1 week thrombosis; vein grafts, arterial conduits or restenotic lesion	1
Wald et al. (9)	465	Immediate CR vs. COR	2008–2013	UK, multi	STEMI with ≥50% stenosis of ≥1 non-IRAs	CS; LM disease; previous CABG; CTO as the only non-IRA; unable to provide consent	1.9 (mean)
Smits et al. (12)	885	Immediate CR vs. COR	2011–2015	8 countries, multi	STEMI with ≥50% stenosis of ≥1 non-IRAs (or their major side branches of ≥ 2.0 mm)	LM disease; CTO; severe stenosis; TIMI flow ≥ 2 in non-IRA; suboptimal result or complications after IRA PCI; severe valve dysfunction; Killip class ≥ III	1
Ghani et al. (7)	121	Staged CR before discharge or within 3 weeks after STEMI vs. COR	2004–2007	Netherlands, single	STEMI with ≥50% stenosis of ≥2 epicardial arteries, or the combination of a side branch and a main epicardial vessel (≥2.5mm)	Urgent indication for additional revascularization; > 80 years old; CTO; previous CABG; LM disease; restenotic lesions in non-IRAs; chronic atrial fibrillation; limited life-expectancy	3
Engstrom et al. (11)	627	Staged CR 2 days after primary PCI before discharge vs. COR	2011–2014	Denmark, multi	STEMI with >50% in ≥1 non-IRAs	Intolerance of contrast media or of relevant anticoagulant or antithrombotic drugs; unconsciousness or CS; ST; indication for CABG; increased bleeding risk	2.3
Hlinomaz et al. (25)	214	Staged CR 3~40 days after primary PCI vs. COR	2009-2013	Czech/Bulgaria, multi	STEMI with ≥70% in ≥1 non-IRAs (≥2.5mm)	CS; LM disease; hemodynamic instability; significant valvular disease; angina pectoris > grade 2 CCS lasting 1 month	3.2
Mehta et al. (18)	4041	Staged CR before discharge or within 45 days after randomization vs. COR	2013-2017	31 countries, multi	STEMI with visually ≥70% or 50~69% with FFR ≤ 0.8 in ≥1 non-IRAs (≥2.5mm)	Intention before randomization to revascularize a nonculprit lesion; planned surgical revascularization; previous CABG; life expectancy <5 year	3
Ochala et al. (23)	92	Immediate CR vs. Staged CR 27 days after primary PCI	NA	Poland, multi	STEMI with >70% in ≥1 non-IRAs	CS; LM disease; previous CABG; target lesion in non-IRA not suitable for PCI; renal insufficiency or presence of 1 kidney; contraindications for antiplatelet therapy; valvular disease requiring surgery	0.5
Maamoun et al. (24)	78	Immediate CR vs. Staged CR within 7 days after primary PCI	2007-2008	Yemen, single	STEMI with ≥70% stenosis of ≥2 angiographically-documented diseased arteries	CS; LM disease; pulmonary edema; previous revascularization; serum creatinine >1.4 mg/dl; contraindication for antiplatelet therapy	1
Tarasov et al. (26)	136	Immediate CR vs. Staged CR 10 days after primary PCI before discharge	2011-2014	Russia, single	STEMI with visually≥70% stenosis of ≥2 epicardial arteries or significant branches (≥2.5mm)	CS; LM disease; contraindication to use heparin, aspirin, clopidogrel, ticagrelor, zotarolimus	1
Politi et al. (6)	214	Immediate CR vs. COR vs. Staged CR 57 days after primary PCI	2003-2007	Italy, single	STEMI with visually>70% stenosis of ≥2 epicardial arteries or their major branches	CS; LM disease; previous CABG; severe valvular disease; unsuccessful procedures	2.5 (mean)

*CABG, coronary artery bypass grafting; CCS, Canadian Cardiovascular Society; CTO, chronic total occlusion; COR, culprit-only revascularization; CR, complete revascularization; CS, cardiogenic shock; IRA, infarct-related artery; LM, left main; NA, not applicable; PCI, percutaneous coronary intervention; STEMI, ST segment elevation myocardial infarction*.

The baseline characteristics of the patients are detailed in Table 2 and Supplementary Table S2. A majority of the participants were middle-aged male with a high prevalence of cardiovascular risk factors. The mean age was 62.0 ± 10.5 years, 79.3% were male, 35.0% had anterior MI, 27.2% had 3-vessel coronary disease, and 81.6% were treated with DES. Weighted analysis showed that baseline characteristics were similar in studies comparing staged vs. immediate complete revascularization. However, less patients had diabetes (14 vs.18%) or were treated with DES (68 vs.78%) in immediate complete revascularization group compared with culprit-only PCI group, while less patients had hypertension (47 vs. 50%) in staged complete revascularization group compared with culprit-only PCI group.

**Table 2:** Baseline characteristics of the patients.

**Study**	**Age, years**	**Male, %**	**Diabetes, %**	**Previous MI, %**	**Smoking, %**	**Hypertension, %**	**Killip class ≥II, %**	**Anterior MI, %**	**Three-vessel disease, %**	**DES use, %**
**Immediate complete revascularization vs. Culprit-only PCI**										
Di Mario et al. (5)	63.9 ± 11.3	87.0	18.8	NA	71.0	42.0	18.8	53.6	34.8	0
Politi et al. (6)	65.6 ± 12.6	76.5	19.5	NA	NA	55.0	NA	44.3	26.8	10.1
Wald et al. (9)	62.0 ± 9.7	78.1	17.8	7.5	47.5	40.2	NA	33.5	35.9	60.6
Smits et al. (12)	61.3 ± 10.0	77.2	15.5	7.9	46.1	47.2	5.1	35.1	32.2	96.9
**Staged complete revascularization vs. Culprit-only PCI**										
Politi et al. (6)	65.5 ± 12.3	77.9	21.5	NA	NA	61.7	NA	42.3	33.6	10.7
Ghani et al. (7)	61.7 ± 10.3	80.2	5.8	5.8	44.6	31.4	5.0	24.0^*^	23.1	20.7
Engstrom et al. (11)	63.5 ± 9.6	80.7	11.3	7.0	49.6	44.0	6.7	34.6	31.4	93.8
Hlinomaz et al. (25)	NA	NA	NA	NA	NA	NA	NA	NA	NA	NA
Mehta et al. (18)	62.0 ± 10.7	79.8	19.5	7.5	39.7	49.7	10.8	34.1^*^	23.4	86.2
**Staged complete revascularization vs. Immediate complete revascularization**										
Ochala et al. (23)	NA	NA	NA	NA	NA	NA	NA	NA	NA	NA
Politi et al. (6)	64.3 ± 11.4	78.5	16.2	NA	NA	56.9	NA	45.4	36.9	8.5
Maamoun et al. (24)	53.5 ± 9.0	92.3	47.4	NA	53.8	35.9	NA	65.4	24.4	33.3
Tarasov et al. (26)	58.9 ± 10.6	66.9	22.1	10.3	NA	91.9	13.2	NA	46.3	100
**Summary**	62.0 ± 10.5	79.3	18.2	7.5	47.1	48.7	9.5	35.0	27.2	81.6

*DES, drug-eluting stent; MI, myocardial infarction; NA, not applicable; PCI, percutaneous coronary intervention*.

* *Culprit vessel of center anterior descending coronary artery*.

### Pairwise Meta-Analysis

**Figure 2:**
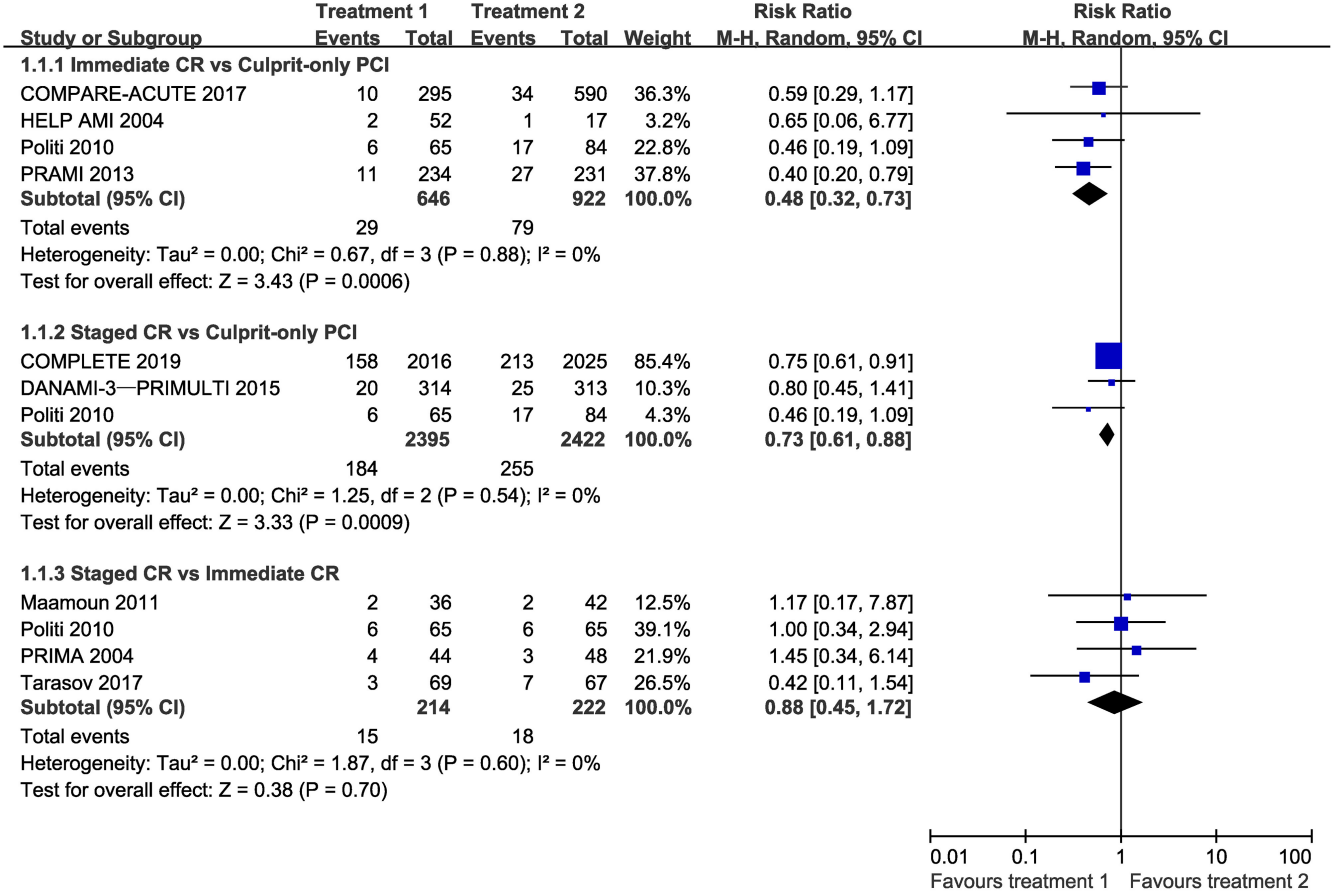
Forest plot of pairwise meta-analysis for cardiovascular mortality or myocardial infarction. CI, confidence interval; CR, complete revascularization; PCI, percutaneous coronary intervention.

Compared with culprit-only PCI, immediate complete revascularization and staged complete revascularization were respectively associated with a 52 and 27% reduction in the risk of cardiovascular death or MI with no evidence of heterogeneity (RR 0.48, 95% CI 0.32–0.73, I^2^ = 0%; and RR 0.73, 95% CI 0.61–0.88, I^2^ = 0%, respectively). However, the incidence of cardiovascular death or MI was not statistically different in staged and immediate complete revascularization groups (RR 0.88, 95% CI 0.45–1.72, I^2^ = 0%) (Figure 2). Trial sequential analysis showed that no significant difference was found between staged and immediate complete revascularization, however, this result may change with an adequate amount of data in the future (Supplementary Figure S2).

As shown in Supplementary Figures S3, S4, the risks of all-cause death and cardiovascular death were similar across the 3 revascularization strategies. Compared with culprit-only PCI, immediate complete revascularization was significantly associated with lower risk of MI without heterogeneity (RR 0.41, 95% CI 0.24–0.70, I^2^ = 0%). However, no statistical differences were found when comparing staged complete revascularization with culprit-only PCI (RR 0.93, 95% CI 0.57–1.49, I^2^ = 47%) or immediate complete revascularization (RR 1.06, 95% CI 0.45–2.50, I^2^ = 0%) with respect to MI (Supplementary Figure S5). Repeat revascularization was significantly reduced by complete revascularization during primary PCI (RR 0.35, 95% CI 0.26–0.48, I^2^ = 0%) or as a staged procedure (RR 0.38, 95% CI 0.17–0.83, I^2^ = 89%) compared with culprit-only PCI. No difference was found between staged complete revascularization and immediate complete revascularization groups with regard to repeat revascularization (RR 1.07, 95% CI 0.64–1.78, I^2^ = 0%) (Supplementary Figure S6). In addition, there was no difference in any of the safety outcomes, i.e., contrast-associated acute kidney injury, stroke, major bleeding and stent thrombosis across the 3 PCI strategies (Supplementary Figures S7–S10).

**Figure 3:**
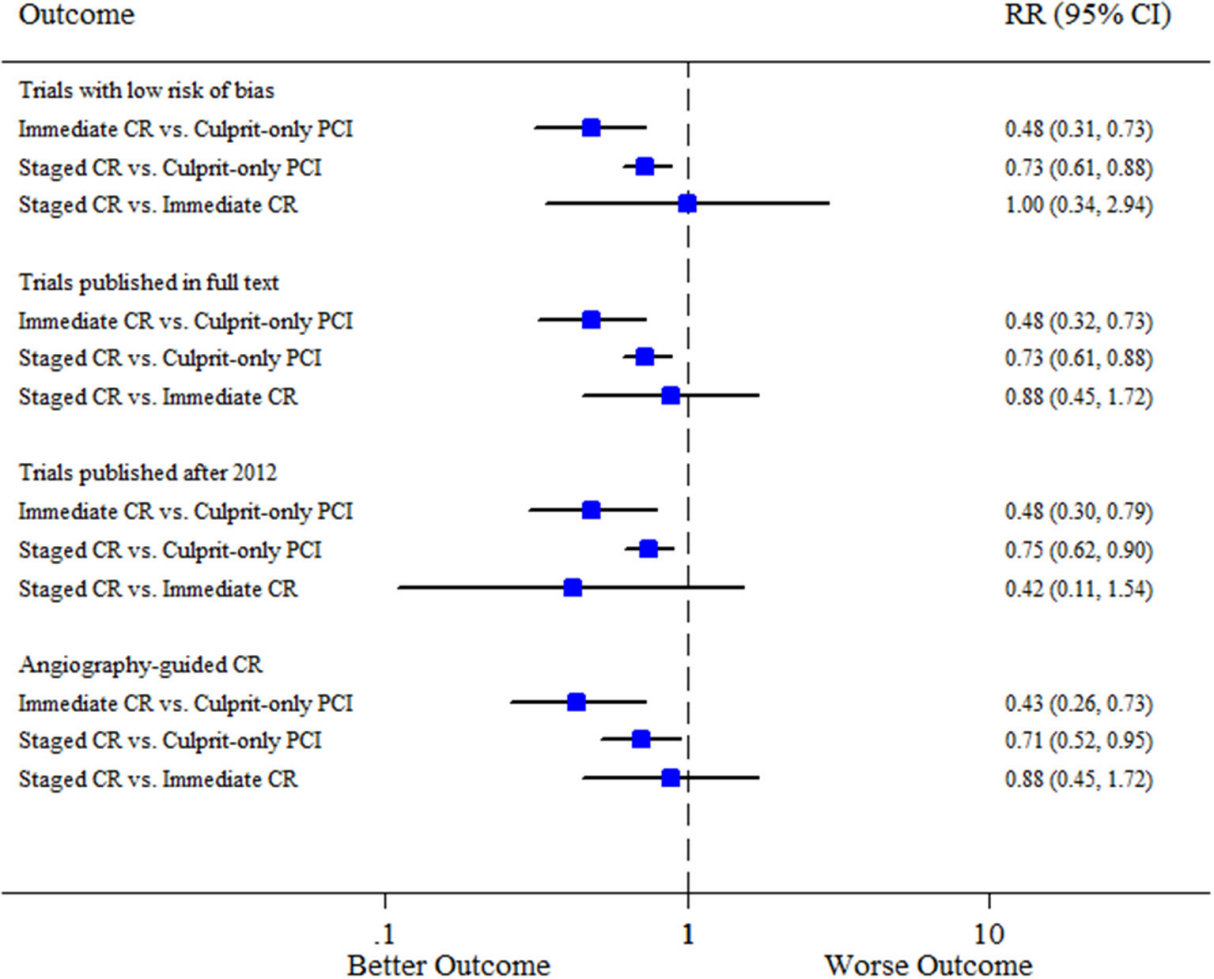
Sensitivity analysis. CI, confidence interval; CR, complete revascularization; PCI, percutaneous coronary intervention; RR, relative risk.

Separate analyses according to the pre-specified variables obtained mostly similar results compared to the overall analysis for all the comparisons (Figure 3). Meta-regression analyses suggested no interaction between the aforementioned covariates and the composite of cardiovascular mortality or MI for all the comparisons (Supplementary Table S3).

### Network Meta-Analysis

**Figure 4:**
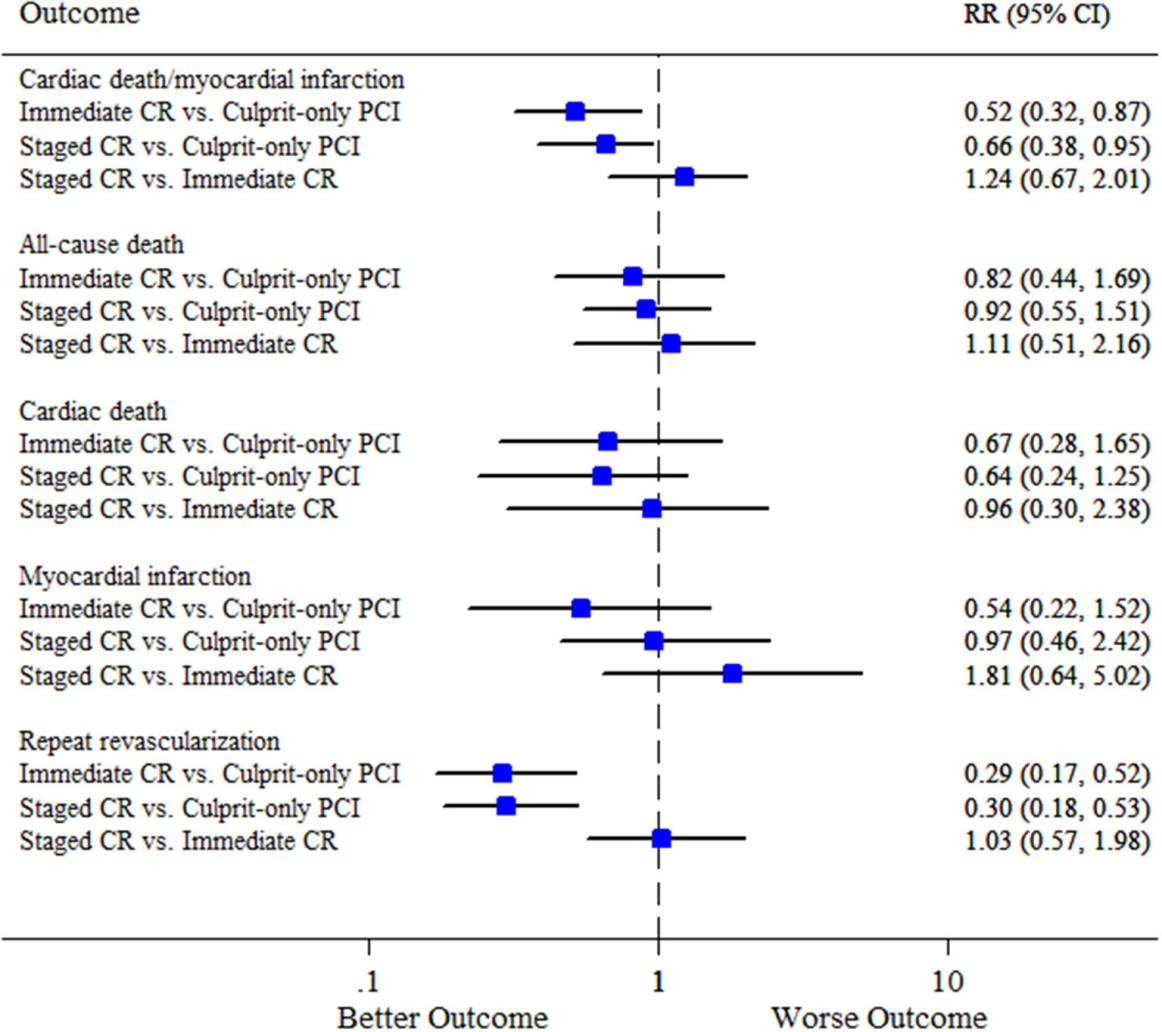
Summary plot for the efficacy outcomes in network meta-analysis. CI, confidence interval; CR, complete revascularization; PCI, percutaneous coronary intervention; RR, relative risk.

We did not find any inconsistencies between evidence from direct and indirect comparisons for all the efficacy endpoints, and the consistency model was consistently applied (Supplementary Table S4). Network meta-analysis was not performed for the safety outcomes due to the limited number of studies included in this analysis. Similar to the results of pairwise meta-analysis, immediate complete revascularization and staged complete revascularization were respectively associated with a 48% and 32% reduction in the risk of cardiovascular death or MI compared with culprit-only PCI (RR 0.52, 95% CI 0.32–0.87; and RR 0.66, 95% CI 0.38–0.95, respectively). The risk of cardiovascular death or MI was not significantly different between staged and immediate complete revascularization (RR 1.24, 95% CI 0.67–2.01) (Figure 4). Rank probability plot noted that complete revascularization during primary PCI had a 77% probability of being the best strategy for reducing cardiovascular death or MI followed by complete revascularization as a staged procedure (Supplementary Figure S11). Additionally, the results of network meta-analysis for the secondary endpoints were almost consistent with pairwise meta-analysis. Note that, immediate complete revascularization was not significantly associated with a reduction in MI compared with culprit-only PCI in network meta-analysis (Figure 4). Funnel plots suggested no publication bias in terms of all the clinical outcomes (Supplementary Figures S12–S20).

## Discussion

This comprehensive meta-analysis of 11 RCTs with 6,942 patients evaluated the relative efficacy and safety of the 3 PCI strategies for hemodynamically stable patients with STEMI and multivessel disease. The principal findings are as follows: (1) both immediate and staged complete revascularization significantly reduced the risks of cardiovascular death or MI and repeat revascularization compared with culprit-only PCI; (2) complete revascularization during primary PCI or as a staged procedure had similar incidence of safety outcomes compared with culprit-only PCI; and (3) No statistical difference was found between immediate and staged complete revascularization in terms of all the outcomes.

The management of non-culprit vessels in STEMI has been the subject of extensive debate for 2 decades. In the past few years (before the COMPLETE trial), several RCTs with moderate sample size highlighted lower risk of repeat revascularization or major adverse cardiovascular events, but not the hard endpoints in patients undergoing immediate or staged multivessel PCI compared with those undergoing culprit-only PCI (9–12). The latest guidelines were hence updated to consider complete revascularization as IIb and IIa recommendations in United States and Europe, reflecting the uncertainty in this field (2, 3). Fortunately, the lack of significant treatment effect of complete revascularization on all-cause death or MI was confirmed by meta-analyses of these trials (13, 14). In addition, meta-analysis performed by Bravo et al. proved that PCI of non-culprit lesions reduced cardiovascular mortality when compared with culprit-only PCI (28). Nevertheless, they combined both immediate and staged multivessel PCI into a single group, which were different in terms of interventional and pathophysiological perspectives (15).

Previous network meta-analysis conducted by Bangalore et al. showed that immediate complete revascularization might be the preferred strategy in patients with STEMI and multivessel disease due to the significantly reduced risks of all-cause death or MI, all-cause death, MI and repeat revascularization, whereas staged complete revascularization only reduced the risk of repeat revascularization, but did not reduce the risk of all-cause death or MI compared with culprit-only PCI (16). Similarly, another network meta-analysis also indicated that immediate complete revascularization was associated with reduction in all-cause mortality and MI compared with culprit-only PCI, while no difference was found between staged complete revascularization and culprit-only PCI for all the endpoints except repeat revascularization (17). In contrast, some network meta-analyses concluded that staged rather than immediate complete revascularization was the best strategy for improving survival in patients with STEMI and multivessel disease (4, 29). However, they mainly included observational studies that could introduce unmeasured confounders and selection bias. In clinical scenarios, clinicians tended to perform single-procedure multivessel PCI in high risk patients with poor cardiac function. Obviously, the relative benefit of immediate complete revascularization, staged complete revascularization, and culprit-only PCI with regard to the hard endpoints in patients with STEMI and multivessel disease remains unclear. The recently published COMPLETE trial with 4,041 patients is the largest study of its kind, and it confirmed that staged PCI of non-culprit vessels reduced the risk of cardiovascular mortality or MI by 26% at 3 years (18). Thus, updated network meta-analysis is needed to compare the relative benefit of the 3 PCI strategies regarding cardiovascular death or MI to support more specific recommendations in guidelines.

Potentially the most important finding of our study is that both immediate and staged complete revascularization can reduce the risk of cardiovascular death or MI compared with culprit-only PCI. One possible explanation of the beneficial effect of complete revascularization is that early PCI of non-culprit lesions can reduce ischemic burden, decrease infarct size and preserve left ventricular function, thereby reducing the risks of heart failure and cardiovascular mortality. In addition, pathophysiological changes such as pro-inflammatory state is generalized in all the arteries rather than in the culprit-vessel alone. Actually, results from the COMPLETE trial optical coherence tomography substudy indicated that 47% of patients who had at least one obstructive non-culprit lesion containing complex vulnerable plaque which were prone to major adverse cardiovascular events (30). Therefore, complete revascularization can timely treat the vulnerable plaques in non-culprit vessel to prevent the subsequent coronary obstruction or embolization, and the progression over time to ischemia and symptoms of angina (31).

Moreover, our meta-analysis based on current RCTs also found that immediate complete revascularization might be ranked as the best revascularization strategy followed by staged complete revascularization for the primary endpoint after the publication of the COMPLETE trial. Note that, staged complete revascularization was consistently not statistically different from immediate complete revascularization for all the outcomes with the currently limited sample size. In fact, the incidence of ischemic events is more common in the first days and then decreases after the first month (1). Compared with immediate multivessel PCI, the delay between index and staged procedures, ranging from 2 to 57 days, may be too long to prevent adverse events. Thus, achieving complete revascularization as soon as possible may help reduce the risk for cardiovascular death and MI in the early phase of STEMI for hemodynamically stable patients. Of note, diagnosis of periprocedural MI in the setting of STEMI is difficult especially in patients undergoing single-procedure multivessel PCI, whereas it can usually be determined in a separate procedure in staged complete revascularization group, thus the incidence of MI might be underestimated in immediate complete revascularization group. In our meta-analysis, only 4 trials including 436 patients that directly compared immediate vs. staged complete revascularization, and no significant difference was found between the two types of multivessel PCI strategies. The ongoing trials (NCT03135275 and NCT03621501) will help to further clarify the optimal timing of complete revascularization in terms of reducing cardiovascular mortality or MI in patients with STEMI and multivessel disease.

In previous guidelines, recommendations for revascularization of non-culprit vessels in STEMI patients were strongly influenced by prolonged intervention time, contrast-induced nephropathy, procedural complications and stent thrombosis in a pro-thrombotic and pro-inflammatory state (8). With the advances in interventional techniques and devices, and the widespread use of new-generation DES and novel antiplatelet therapy, complete revascularization is increasingly considered as a safe and feasible approach for patients with STEMI and multivessel disease. Unsurprisingly, we did not observe a significant increase in the risks of contrast-induced nephropathy, stroke, major bleeding and stent thrombosis in immediate or staged complete revascularization strategy, compared with culprit-only PCI in the present analysis.

## Limitations

Our study presents several limitations that cannot be ignored. First, as a trial-level meta-analysis, this study was limited by variability in inclusion or exclusion criteria, procedural technique, stent type, timing of staged PCI, and follow-up duration. Therefore, random-effects model was used all across the study and sensitivity analyses yielded mostly similar results compared to the overall results. Furthermore, meta-regression analyses did not find any significant factor that contributed to the heterogeneity. Second, the results should be extrapolated to high-risk patients carefully. All RCTs included only hemodynamically stable patients, and patients with cardiogenic shock, left main disease, and CTO in non-culprit vessels were specifically excluded in most of the trials. Third, our study did not have enough data to suggest the optimal timing of staged complete revascularization, i.e., during hospitalization or within a few weeks after STEMI. Fourth, we did not evaluate whether the determination of non-culprit lesions requiring PCI should be guided by angiography or FFR, as FFR was mainly applied in only 3 trials. The ongoing trials will contribute to specify the better approach to guide the revascularization of non-culprit vessels (NCT02862119, NCT02637440, NCT03562572, NCT02715518, and NCT03772743). Fifth, blinding was not possible for the included trials, the knowledge that patients had untreated significant non-culprit lesions may by itself drive the incidence of new revascularizations in culprit-only PCI group.

## Conclusions

The current evidence suggests that complete revascularization during primary PCI or as a staged procedure is safe and feasible for hemodynamically stable patients with STEMI and multivessel disease. Both immediate and staged complete revascularization were associated with a reduction of cardiovascular death or MI compared with culprit-only PCI. According to trial sequential analysis, further trials are warranted to directly compare immediate vs. staged complete revascularization in terms of cardiovascular mortality or MI.

## Data Availability Statement

The original contributions presented in the study are included in the article/Supplementary Material, further inquiries can be directed to the corresponding author/s.

## Author Contributions

KD and KC contributed to the study design and interpretation of the results. DY, LF, CZ, SY, and SW collected the data. KC, DY, and LF analyzed the data. KC prepared the manuscript. LF, KD, DY, and CZ revised the manuscript. All authors contributed to the article and approved the submitted version.

## Funding

The current study was funded by CAMS Innovation Fund for Medical Sciences (CIFMS) (2021-I2M-1-008) and Beijing Municipal Health Commission-Capital Health Development Research Project (2020-1-4032).

## Conflict of Interest

The authors declare that the research was conducted in the absence of any commercial or financial relationships that could be construed as a potential conflict of interest.

## Publisher's Note

All claims expressed in this article are solely those of the authors and do not necessarily represent those of their affiliated organizations, or those of the publisher, the editors and the reviewers. Any product that may be evaluated in this article, or claim that may be made by its manufacturer, is not guaranteed or endorsed by the publisher.
