# Supplementary material for: Optimal Revascularization Strategy for Patients With ST-segment Elevation Myocardial Infarction and Multivessel Disease: A Pairwise and Network Meta-Analysis
Source: Front Cardiovasc Med. 2022 Jan 5;8:695822. doi: 10.3389/fcvm.2021.695822 (PMC8767564; doi:10.3389/fcvm.2021.695822)
Supplement: Supplementary file 1 [file Data_Sheet_1.docx]

**Supplementary Appendix**

**Optimal revascularization strategy for patients with ST-segment elevation myocardial infarction and multivessel disease: A pairwise and network meta-analysis**

Table S1 Definition of outcomes. Pages 3-4

Table S2 Baseline characteristics of the patients in each group. Pages 5-6

Table S3 Assessment of inconsistency beween the direct and indirect evidence. Page 7

Table S4 Meta-regression analysis. Page 8

Figure S1 The risk of bias of each trial according to the Cochrane Collaboration’s tool. Page 9

Figure S2 Trial sequential analysis of cardiac death or myocardial infarction. Page 10-11

Figure S3 Forest plot of pairwise meta-analysis for all-cause mortality. Page 12

Figure S4 Forest plot of pairwise meta-analysis for cardiovascular mortality. Page 13

Figure S5 Forest plot of pairwise meta-analysis for myocardial infarction. Page 14

Figure S6 Forest plot of pairwise meta-analysis for repeat revascularization. Page 15

Figure S7 Forest plot of pairwise meta-analysis for contrast-associated acute kidney injury. Page 16

Figure S8 Forest plot of pairwise meta-analysis for stroke. Page 17

Figure S9 Forest plot of pairwise meta-analysis for major bleeding. Page 18

Figure S10 Forest plot of pairwise meta-analysis for stent thrombosis. Page 19

Figure S11 Rank probability analysis results of all the efficacy outcomes. Pages 20-22

Figure S12 Funnel plot of studies for the risk of cardiovascular mortality or myocardial infarction. Page 23

Figure S13 Funnel plot of studies for the risk of all-cause mortality. Page 24

Figure S14 Funnel plot of studies for the risk of cardiovascular mortality. Page 25

Figure S15 Funnel plot of studies for the risk of myocardial infarction. Page 26

Figure S16 Funnel plot of studies for the risk of repeat revascularization. Page 27

Figure S17 Funnel plot of studies for the risk of contrast-associated acute kidney injury. Page 28

Figure S18 Funnel plot of studies for the risk of stroke. Page 29

Figure S19 Funnel plot of studies for the risk of major bleeding. Page 30

Figure S20 Funnel plot of studies for the risk of stent thrombosis. Page 31

**Table S1 Definition of Outcomes.**

| Study | Definitions |
| --- | --- |
| HELP AMI 2004 | Not reported. |
| PRAMI 2013 | **Myocardial infarction:** Symptoms of cardiac ischemia and a troponin level above the 99th centile. For patients with a recurrent myocardial infarction within 14 days after randomization, the definition required new electrocardiographic evidence of ST-segment elevation or left bundle branch block and angiographic evidence of coronary artery occlusion.  **Major bleeding:** Bleeding requiring transfusion or surgery.  **Contrast-induced nephropathy:** Requiring dialysis. |
| COMPARE-ACUTE 2017 | **Repeat revascularization:** All recurrent revascularizations were evaluated by the clinical evaluation committee for both the extent of need (urgent or elective) and indication (clinically indicated or not). Clinically indicated elective revascularizations performed within 45 days after primary PCI were not counted as events in the group receiving PCI for an infarct-related coronary artery only. |
| Ghani 2012 | **Myocardial infarction:** New Q-waves on the electrocardiogram or a new CK and CK-MB rise above the upper limit of normal.  **Major bleeding: N**eed for transfusion and/or surgical therapy. |
| DANAMI-3—PRIMULTI 2015 | **Cardiac death:** We judged all deaths cardiac-related unless they could be clearly attributed to another cause, as determined by the clinical events committee.  **Myocardial infarction:** We defined re-infarction when typical chest pain was accompanied by a substantial rise in troponins, development of new Q-waves on the electrocardiograph, or both.  **Repeat revascularization:** Ischemia-driven (subjective or objective) revascularization.  **Contrast-induced nephropathy:** >50% rise in plasma creatinine.  **Major bleeding:** Bleeding requiring transfusion or surgery. |
| PRAGUE-13 2015 | Not reported. |
| COMPLETE 2019 | **Cardiac death:** All deaths with a clear cardiovascular or unknown cause, will be classified as cardiovascular. Only deaths due to a documented non-cardiovascular cause (e.g., cancer) will be classified as non-cardiovascular.  **Myocardial infarction:** Defined according to the third universal definition and was sub-classified according to type.  **Repeat revascularization:** Ischemic symptoms consistent with Canadian Cardiovascular Society class ≥2 angina despite optimal medical therapy, and PCI or CABG of either the culprit lesion (within 5 mm of the stented segment) associated with the index PCI or a non-culprit lesion that led to enrollment into the trial, and At least one of the following: (1) Positive functional study; (2) New ischemic electrocardiogram changes at rest or with exertion in a distribution consistent with a stenosis; (3) Fractional flow reserve ≤0.80.  **Major Bleeding:** Clinically overt, symptomatic bleeding with at least one of the following criteria: (1) Fatal; (2) Symptomatic intracranial hemorrhage; (3) Retroperitoneal hemorrhage; (4) Intraocular hemorrhage leading to significant vision loss; (5) Decrease in hemoglobin of 3.0 g/dL (with each blood transfusion unit counting for 1.0 g/dL of Hb) or requiring transfusion of two or more units of red blood cells or equivalent of whole blood (6) Requiring surgical intervention to stop the bleeding.  **Stroke:** Defined as the presence of a new focal neurologic deficit thought to be vascular in origin, with signs or symptoms lasting more than 24 hours. It is strongly recommended (but not required) that an imaging procedure such as computer tomography scan or magnetic resonance imaging be performed. |
| PRIMA 2004 | Not reported. |
| Maamoun 2011 | **Cardiac death:** If death was caused by fatal arrhythmia, heart failure, newly occurred myocardial infarction, or sudden cardiac death.  **Contrast induced nephropathy:** An absolute increase in serum creatinine values of ≥ 0.5 mg/dl or a ≥ 25% relative increase from baseline within 72 hours following the procedure.  **Major bleeding:** Requires surgical intervention and/or blood transfusion.  **Stroke**: Any neurologic event whether hemorrhagic or non-hemorrhagic stroke. |
| Tarasov 2017 | **Repeat revascularization:** PCI or CABG with the reason for their implementation such as recurrent symptoms, re-infarction or significant ischemia on stress-testing. In staged complete revascularization group, re-revascularization was only unplanned PCI or CABG.  **Myocardial infarction:** Criteria of re-infarction during 18 hours after index myocardial infarction was myocardial ischemia with appropriate recurrent ST-segment deviation ≥30 minutes. After 18 hours re-infarction was diagnosed in the case of new Q-waves, new left bundle-branch block, and/or appropriate increasing of the level of troponin and/or CK-MB fraction. |
| Politi 2010 | **Repeat revascularization:** All PCI or CABG occurring after the baseline procedure and justified by recurrent symptoms, re-infarction or objective evidence of significant ischemia on provocative testing. Among repeat PCI we excluded staged procedures already scheduled. In the staged group we classified as repeat revascularization only unplanned procedures.  **Contrast-induced nephropathy**: An absolute increase in serum creatinine values of 0.5 mg/dl or greater or a 25% or greater relative increase from baseline within 72 hours following both primary and elective PCI. |

CABG = coronary artery bypass grafting; CK-MB = creatine kinase-myocardial band; PCI = percutaneous coronary intervention.

**Table S2 Baseline Characteristics of the Patients in Each Group.**

| Study | Age, years | Male, % | Diabetes, % | Previous MI, % | Smoking, % | Hypertension, % | Killip class ≥II, % | Anterior MI, % | 3-vessel disease, % | DES use, % |
| --- | --- | --- | --- | --- | --- | --- | --- | --- | --- | --- |
| **Immediate complete revascularization vs. Culprit-only PCI** | | | | | | | | | | |
| HELP AMI 2004 | 64±12 vs.65±7 | 88 vs.85 | 12 vs.41 | NA | 67 vs.81 | 37 vs.59 | 20 vs.19 | 52 vs.59 | 31 vs.47 | 0 vs.0 |
| Politi 2010 | 65±12 vs.67±13 | 77 vs.76 | 14 vs.24 | NA | NA | 49 vs.60 | NA | 48 vs.42 | 29 vs.25 | 8 vs 12 |
| PRAMI 2013 | 62±10 vs.62±10 | 76 vs.81 | 15 vs.21 | 8 vs.7 | 50 vs.45 | 40 vs.40 | NA | 29 vs.39 | 39 vs.33 | 63 vs.58 |
| COMPARE-ACUTE 2017 | 62±10 vs.61±10 | 79 vs.76 | 15 vs.16 | 7 vs.8 | 41 vs.49 | 46 vs.48 | 5 vs.5 | 36 vs.35 | 31 vs.33 | 97 vs.97 |
| Summary | 62±10 vs.62±10 | 78 vs.77 | **14 vs.18** | 8 vs.8 | 47 vs.48 | 43 vs.47 | 7 vs.5 | 36 vs.37 | 34 vs.32 | **68 vs.78** |
| **Staged complete revascularization vs. Culprit-only PCI** | | | | | | | | | | |
| Politi 2010 | 64±11 vs.67±13 | 80 vs.76 | 18 vs.24 | NA | NA | 65 vs.60 | NA | 44 vs.42 | 45 vs.25 | 9 vs.12 |
| Ghani 2012 | 62±10 vs.61±11 | 80 vs.80 | 6 vs.5 | 6 vs.5 | 44 vs.48 | 26 vs. 43 | 2 vs.2 | 21 vs.29***** | 25 vs.20 | 23 vs.17 |
| DANAMI-3—PRIMULTI 2015 | 64±10 vs.63±10 | 80 vs.81 | 9 vs.13 | 5 vs.9 | 51 vs.48 | 41 vs.47 | 7 vs.6 | 33 vs.36 | 31 vs.32 | 95 vs.93 |
| PRAGUE-13 2015 | NA | NA | NA | NA | NA | NA | NA | NA | NA | NA |
| COMPLETE 2019 | 62±11 vs.62±11 | 81 vs.79 | 19 vs.20 | 7 vs.8 | 41 vs.39 | 49 vs.51 | 11 vs.11 | 34 vs.34***** | 24 vs.23 | 86 vs.86 |
| Summary | 62±11 vs.62±11 | 80 vs.79 | 17 vs.19 | 7 vs.8 | 42 vs.40 | **47 vs.50** | 10 vs.10 | 34 vs.34 | 25 vs.24 | 83 vs.83 |
| **Staged complete revascularization vs. Immediate complete revascularization** | | | | | | | | | | |
| PRIMA 2004 | NA | NA | NA | NA | NA | NA | NA | NA | NA | NA |
| Politi 2010 | 64±11 vs.65±12 | 80 vs.77 | 18 vs.14 | NA | NA | 65 vs.49 | NA | 44 vs.48 | 45 vs.29 | 9 vs.8 |
| Maamoun 2011 | 52±7 vs.55±10 | 89 vs.95 | 56 vs.40 | NA | 56 vs.52 | 33 vs.38 | NA | 70 vs.62 | 22 vs.26 | 32 vs.36 |
| Tarasov 2017 | 59±11 vs.59±10 | 62 vs.72 | 20 vs.24 | 6 vs.15 | NA | 88 vs.94 | 12 vs.15 | NA | 45 vs.48 | 100 vs.100 |
| Summary | 60±11 vs.60±11 | 75 vs.79 | 27 vs.24 | 6 vs.15 | 56 vs.52 | 68 vs.64 | 12 vs.15 | 52 vs.53 | 40 vs.36 | 51 vs.50 |

DES = drug-eluting stent; MI = myocardial infarction; NA = not applicable; PCI = percutaneous coronary intervention.

*****Culprit vessel of left anterior descending coronary artery.

**Table S3 Meta-Regression Analysis.**

| Patient Characteristics | Regression Coefficient (95% Confidence Interval) | | |
| --- | --- | --- | --- |
|  | ICR vs. COR | SCR vs. COR | SCR vs. ICR |
| Age | -0.027 (-0.569 to 0.514) | -0.089 (-1.529 to 1.351) | 0.017 (-1.343 to 1.377) |
| Male | 0.013 (-0.496 to 0.522) | 0.196 (-2.211 to 2.602) | 0.045 (-0.533 to 0.624) |
| Diabetes | -0.073 (-0.658 to 0.513) | -0.018 (-0.489 to 0.454) | 0.009 (-0.473 to 0.491) |
| Hypertension | 0.010 (-0.151 to 0.171) | -0.031 (-0.412 to 0.350) | -0.021 (-0.264 to 0.222) |
| Anterior MI | 0.006 (-0.170 to 0.183) | **-**0.060 (-0.766 to 0.647) | NA |
| Three-vessel disease | -0.013 (-0.278 to 0.251) | -0.015 (-0.397 to 0.367) | -0.052 (-0.728 to 0.624) |
| Use of DES | 0.002 (-0.024 to 0.029) | 0.007 (-0.068 to 0.082) | -0.010 (-0.129 to 0.110) |
| Follow-up duration | -0.229 (-1.766 to 1.307) | 0.089 (-6.667 to 6.844) | 0.364 (-6.231 to 6.960) |

COR = culprit-only revascularization; DES = drug-eluting stent; ICR = immediate complete revascularization; MI = myocardial infarction; NA = not applicable; SCR = staged complete revascularization.

**Table S4 Assessment of Inconsistency Between the Direct and Indirect Evidence.**

| Outcome | Comparison | Log RR (95% CI) | | | p value |
| --- | --- | --- | --- | --- | --- |
|  |  | Direct Evidence | Indirect Evidence | Network Evidence |  |
| Cardiovascular death or myocardial infarction | COR vs ICR | -0.84 (-1.31 to -0.27) | -0.13 (-1.18 to 0.87) | -0.65 (-1.14 to -0.14) | 0.22 |
|  | COR vs SCR | -0.35 (-0.89 to 0.03) | -1.02 (-2.12 to 0.03) | -0.42 (-0.97 to -0.05) | 0.25 |
|  | ICR vs SCR | -0.19 (-0.93 to 0.60) | 0.50 (-0.21 to 1.26) | 0.21 (-0.40 to 0.70) | 0.15 |
| All-cause death | COR vs ICR | -0.29 (-1.03 to 0.44) | 0.19 (-1.54 to 2.18) | -0.20 (-0.81 to 0.52) | 0.58 |
|  | COR vs SCR | -0.06 (-0.56 to 0.51) | -0.57 (-2.46 to 1.25) | -0.09 (-0.59 to 0.42) | 0.58 |
|  | ICR vs SCR | -0.47 (-1.52 to 0.58) | 0.44 (-0.38 to 1.41) | 0.10 (-0.67 to 0.77) | 0.14 |
| Cardiovascular death | COR vs ICR | -0.52 (-1.51 to 0.47) | -0.09 (-2.37 to 2.23) | -0.40 (-1.26 to 0.50) | 0.73 |
|  | COR vs SCR | -0.42 (-1.51 to 0.32) | -0.88 (-3.46 to 1.37) | -0.44 (-1.41 to 0.22) | 0.71 |
|  | ICR vs SCR | -0.67 (-2.11 to 0.65) | 0.40 (-1.03 to 1.59) | -0.04 (-1.21 to 0.87) | 0.20 |
| Myocardial infarction | COR vs ICR | -1.02 (-2.14, 0.11) | 0.37 (-1.26, 2.29) | -0.62 (-1.50, 0.42) | 0.14 |
|  | COR vs SCR | 0.15 (-0.58, 1.25) | -1.23 (-3.10, 0.63) | -0.03 (-0.78, 0.88) | 0.13 |
|  | ICR vs SCR | 0.07 (-1.22, 1.40) | 1.23 (-0.10, 3.11) | 0.59 (-0.44, 1.61) | 0.17 |
| Repeat Revascularization | COR vs ICR | -1.27 (-1.95, -0.60) | -1.13 (-2.33, 0.10) | -1.23 (-1.78, -0.65) | 0.83 |
|  | COR vs SCR | -1.19 (-1.81, -0.49) | -1.31 (-2.53, -0.08) | -1.21 (-1.72, -0.63) | 0.84 |
|  | ICR vs SCR | 0.02 (-0.83, 0.87) | 0.09 (-0.83, 1.16) | 0.03 (-0.56, 0.68) | 0.90 |

CI = confidence interval; COR = culprit-only revascularization; ICR = immediate complete revascularization; RR = relative risk; SCR = staged complete revascularization.


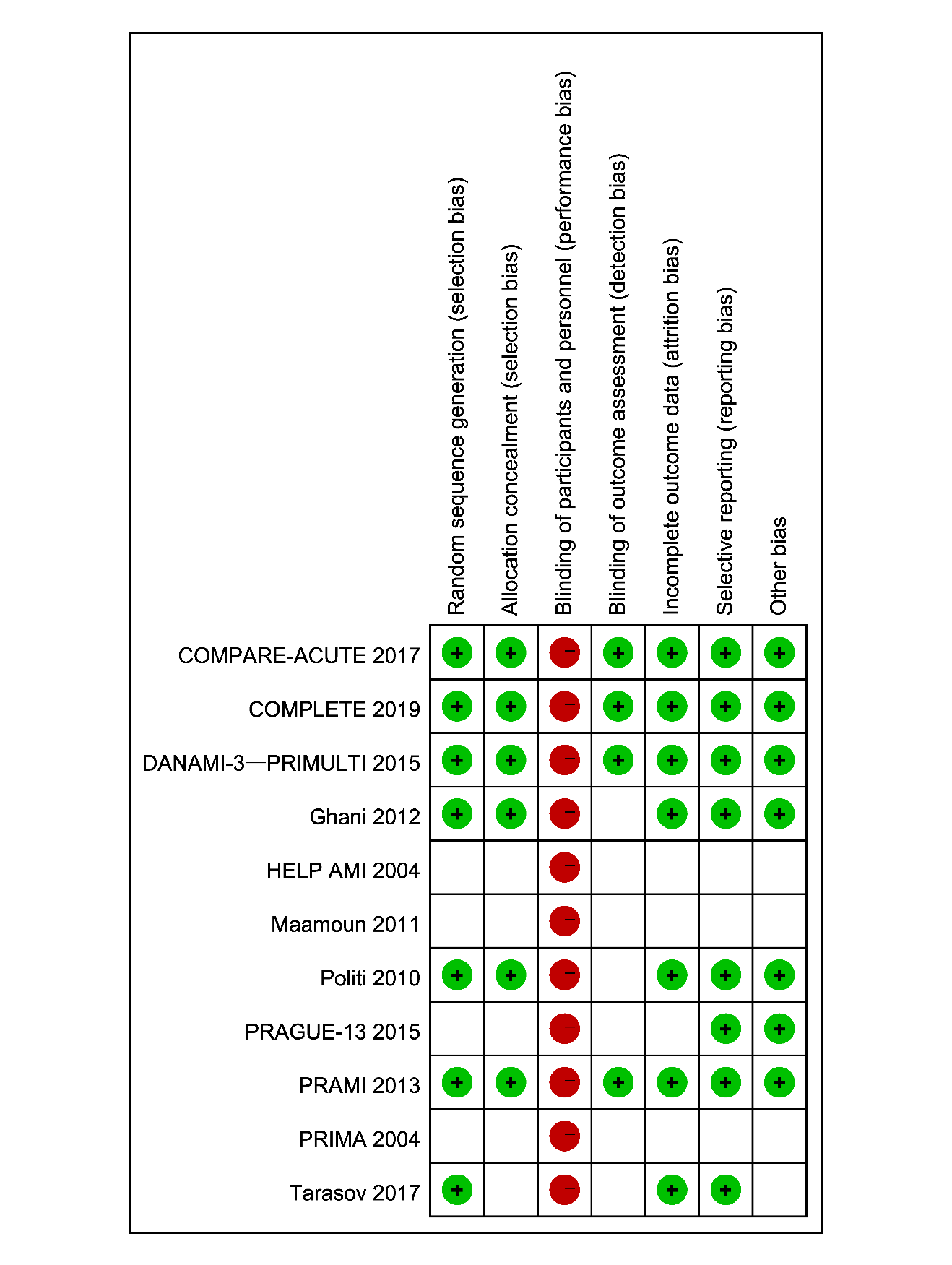
**Figure S1 The Risk of Bias of Each Trial According to the Cochrane Collaboration’s Tool.**

**Figure S2 Trial sequential analysis of cardiac death or myocardial infarction. (A) Immediate CR vs. culprit-only PCI; (B) Staged CR vs. culprit-only PCI; (C) Immediate CR vs. Staged CR.** CR=complete revascularization; PCI=percutaneous coronary intervention.

**
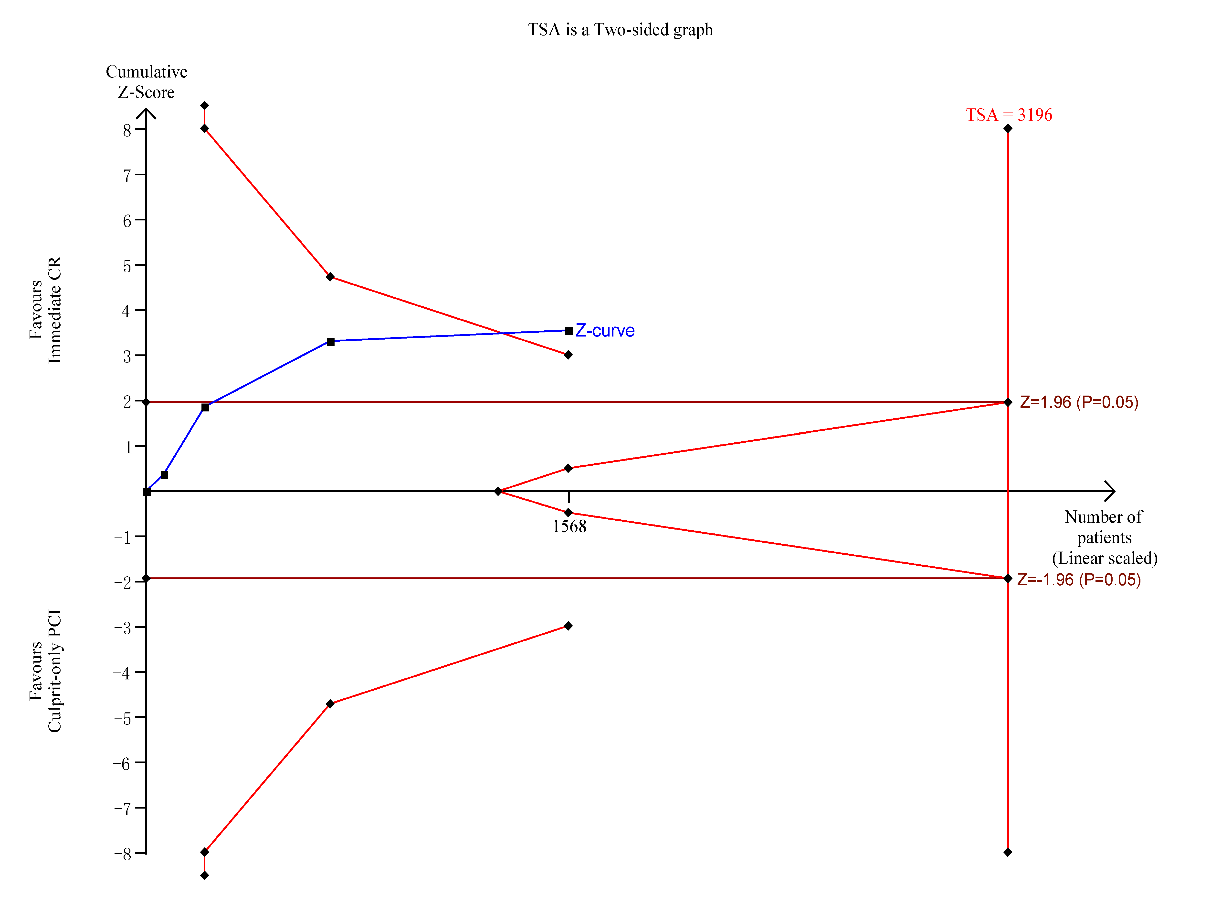
(A)**

**
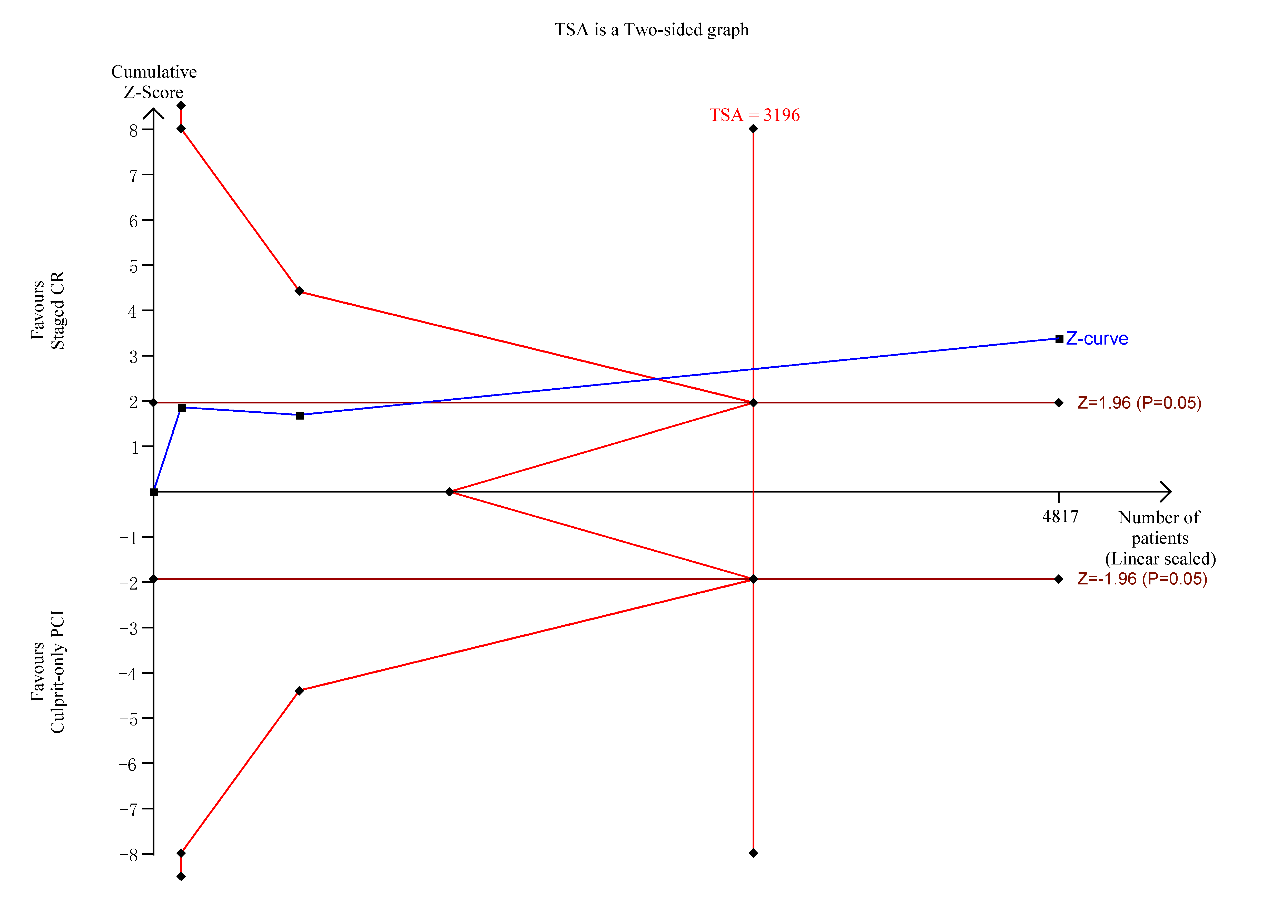
(B)**

**
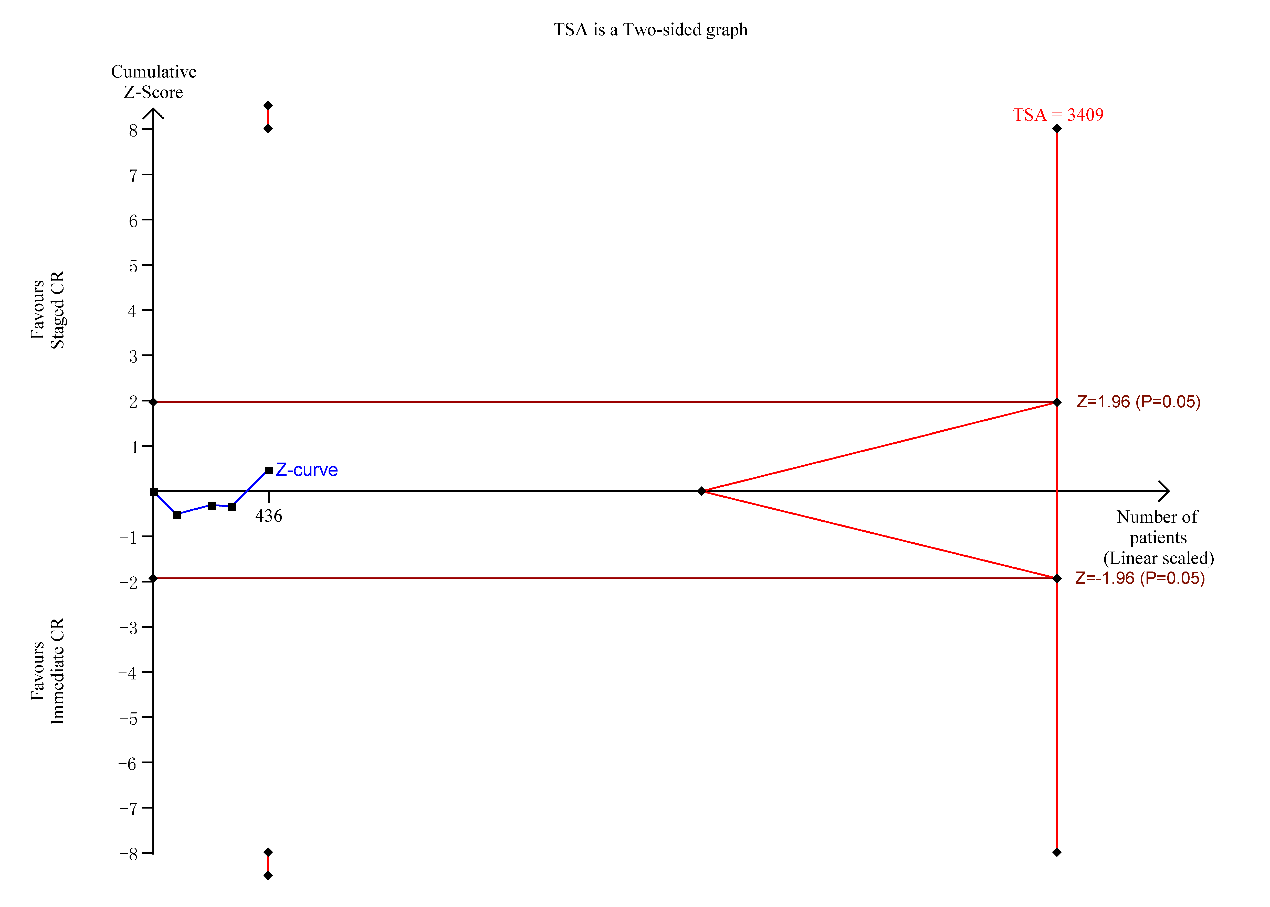
(C)**


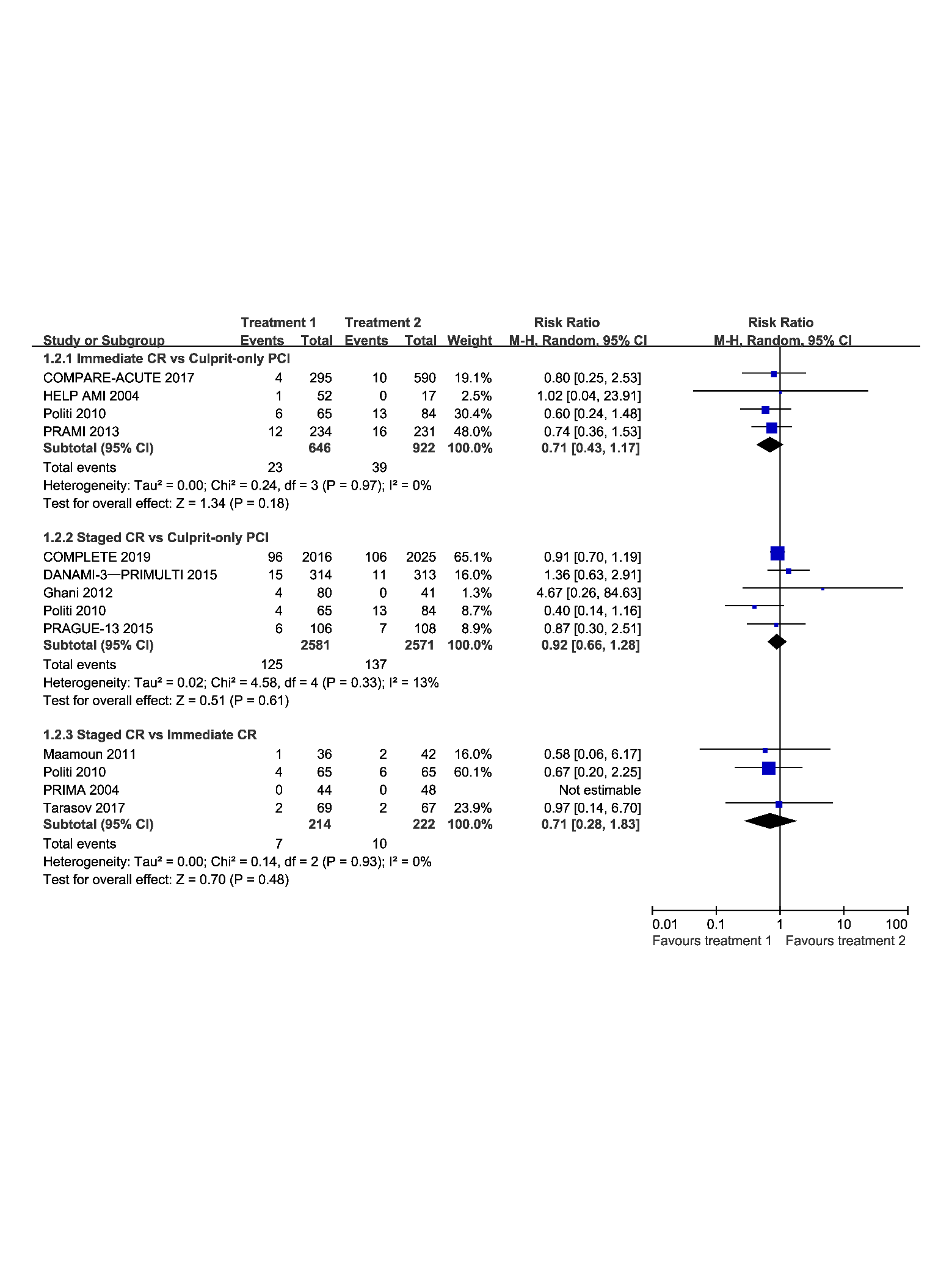
**Figure S3 Forest Plot of Pairwise Meta-Analysis for All-Cause Death.** CI=confidence interval; CR=complete revascularization; PCI=percutaneous coronary intervention.


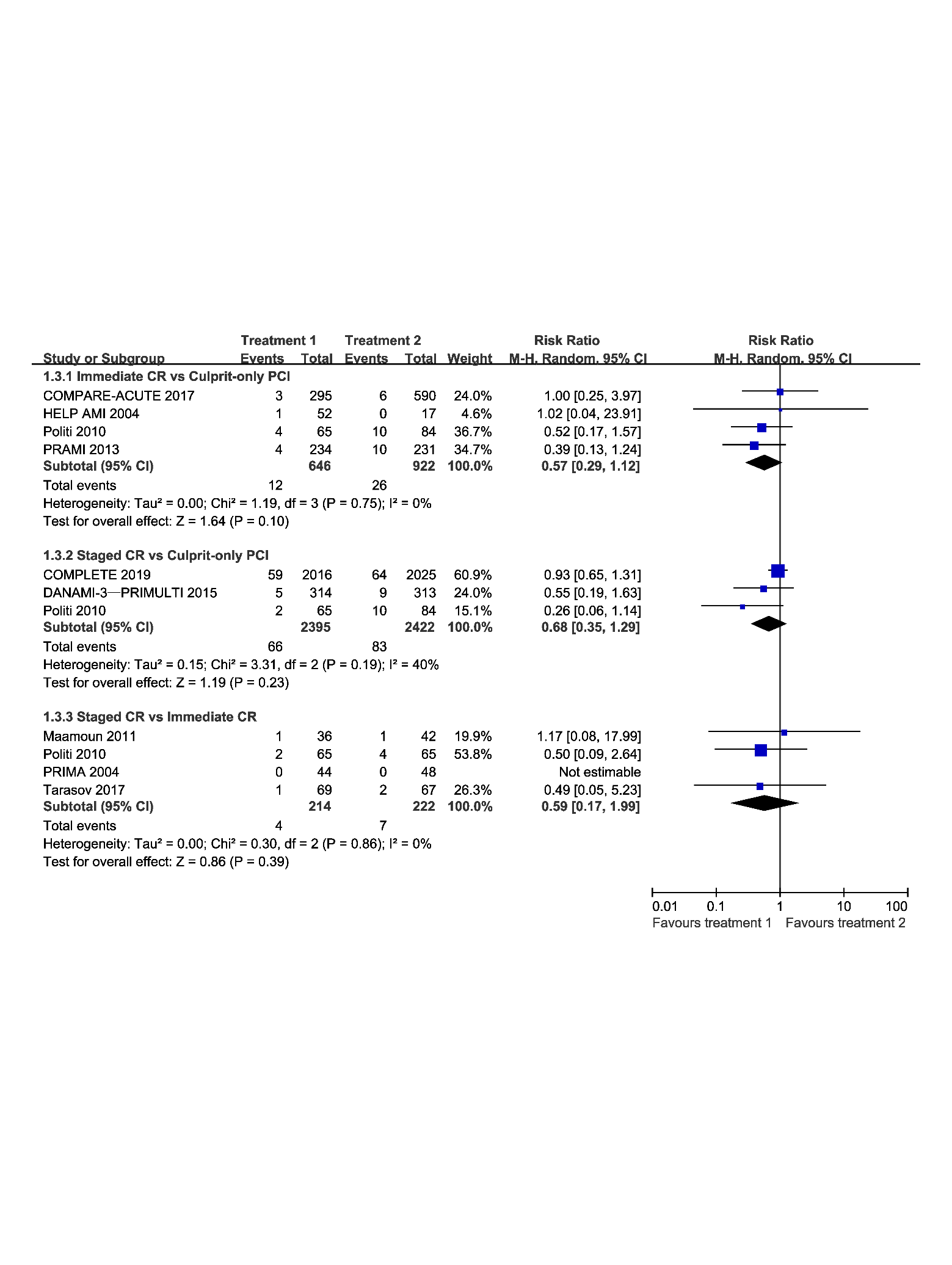
**Figure S4 Forest Plot of Pairwise Meta-Analysis for Cardiac Death.** CI=confidence interval; CR=complete revascularization; PCI=percutaneous coronary intervention.


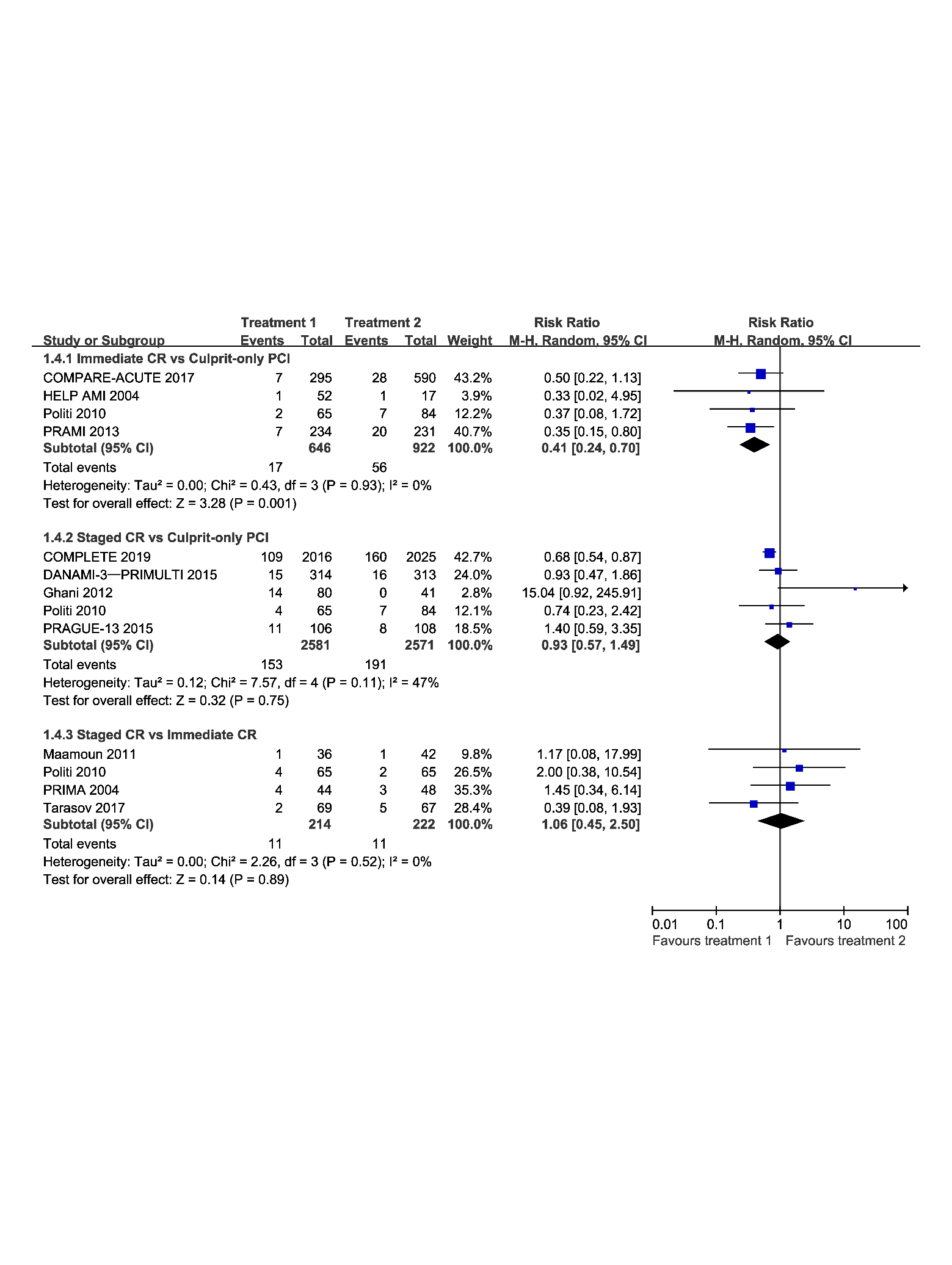
**Figure S5** **Forest Plot of Pairwise Meta-Analysis for Myocardial Infarction.** CI=confidence interval; CR=complete revascularization; PCI=percutaneous coronary intervention.


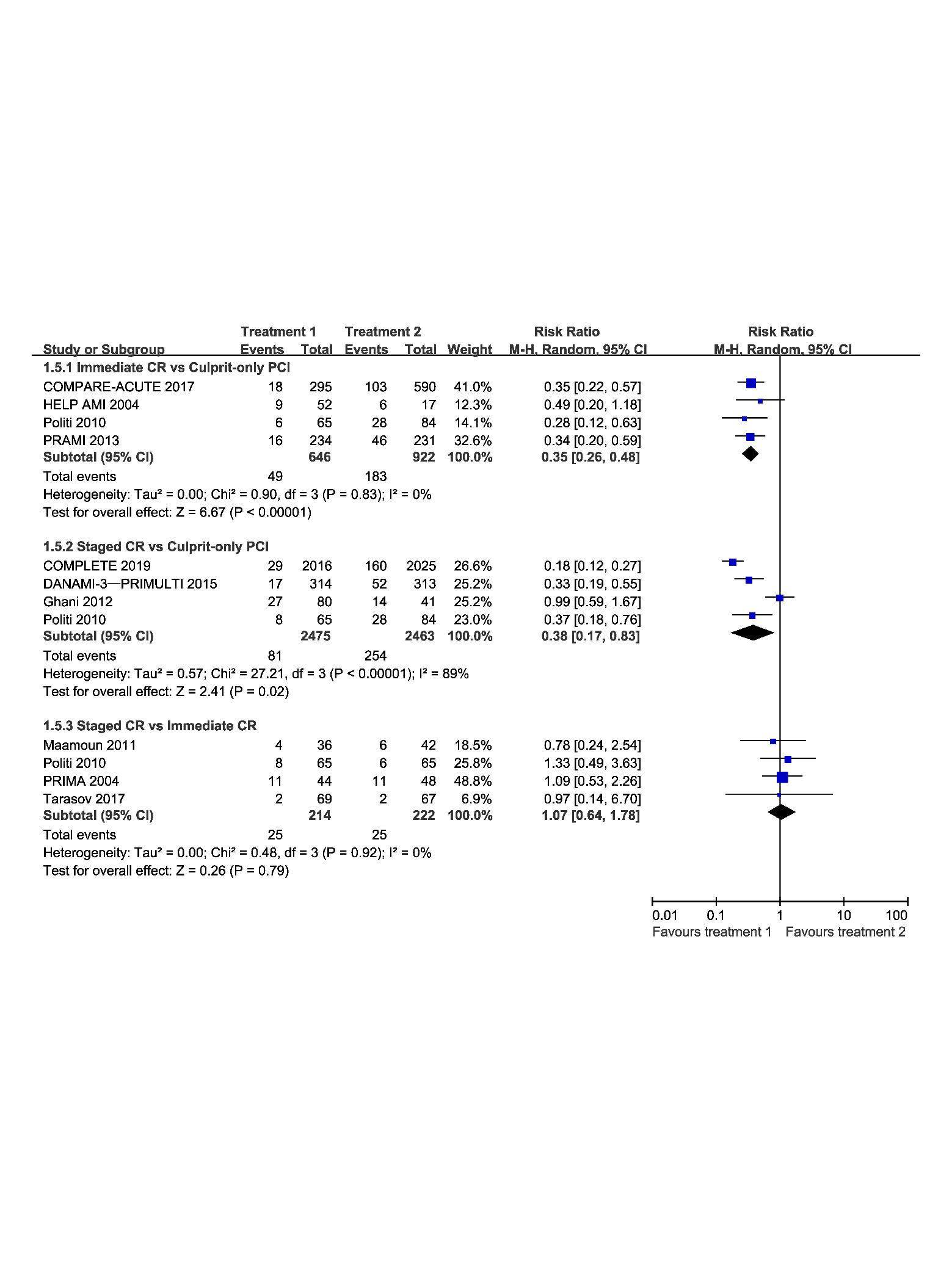
**Figure S6** **Forest Plot of Pairwise Meta-Analysis for Repeat Revascularization.** CI=confidence interval; CR=complete revascularization; PCI=percutaneous coronary intervention.


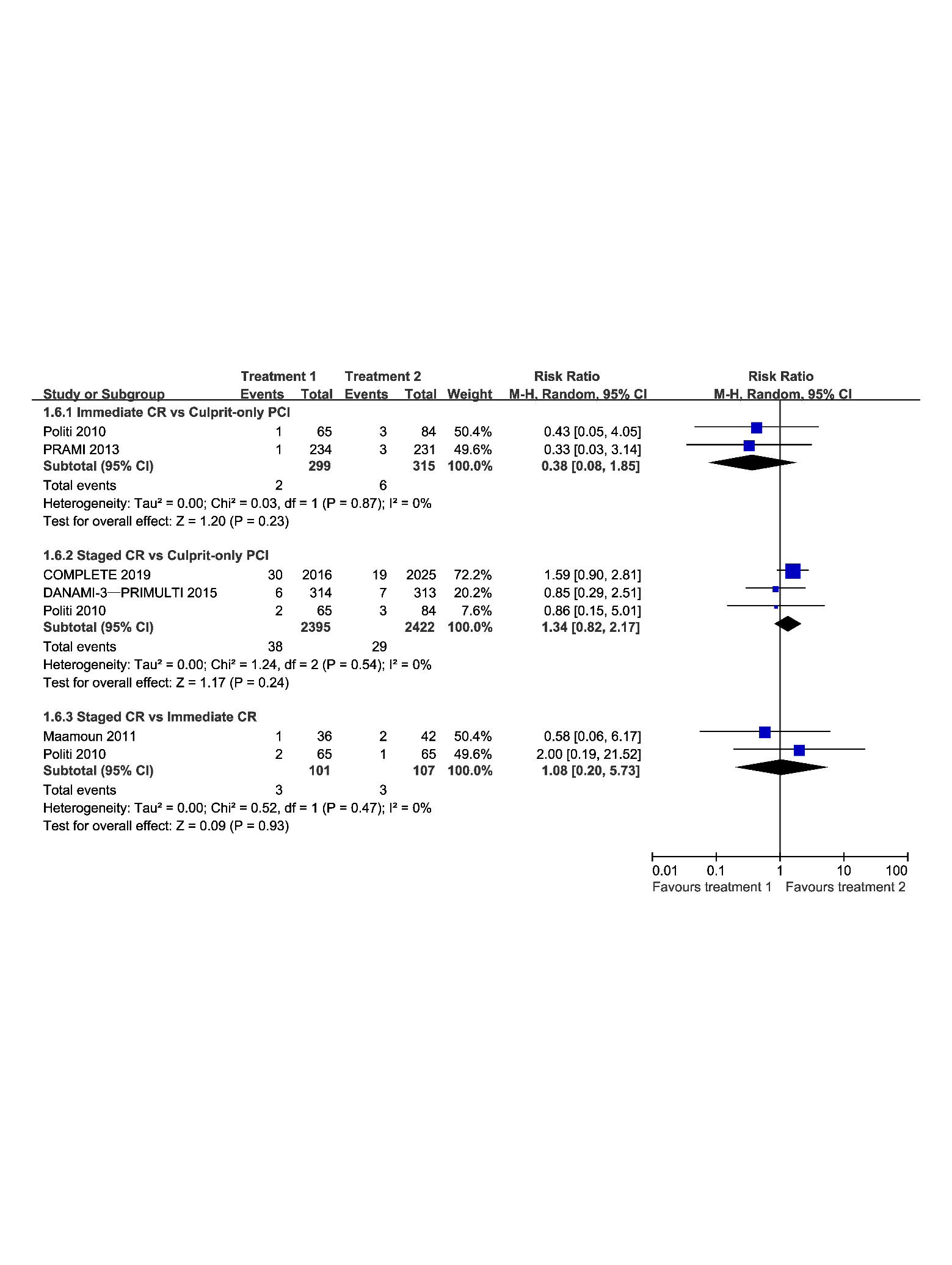
**Figure S7** **Forest Plot of Pairwise Meta-Analysis for Contrast-Associated Acute Kidney Injury.** CI=confidence interval; CR=complete revascularization; PCI=percutaneous coronary intervention.


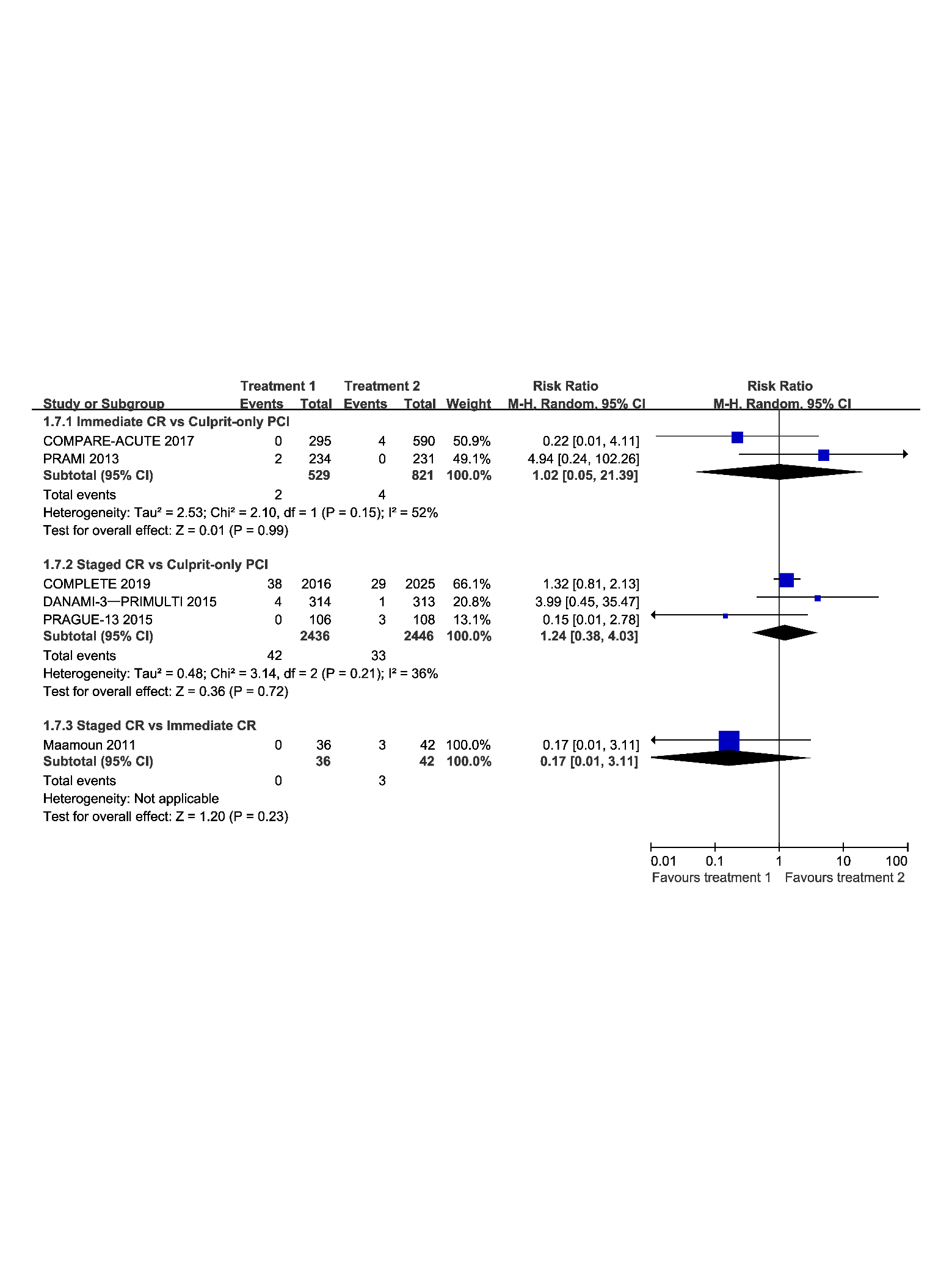
**Figure S8** **Forest Plot of Pairwise Meta-Analysis for Stroke.** CI=confidence interval; CR=complete revascularization; PCI=percutaneous coronary intervention.


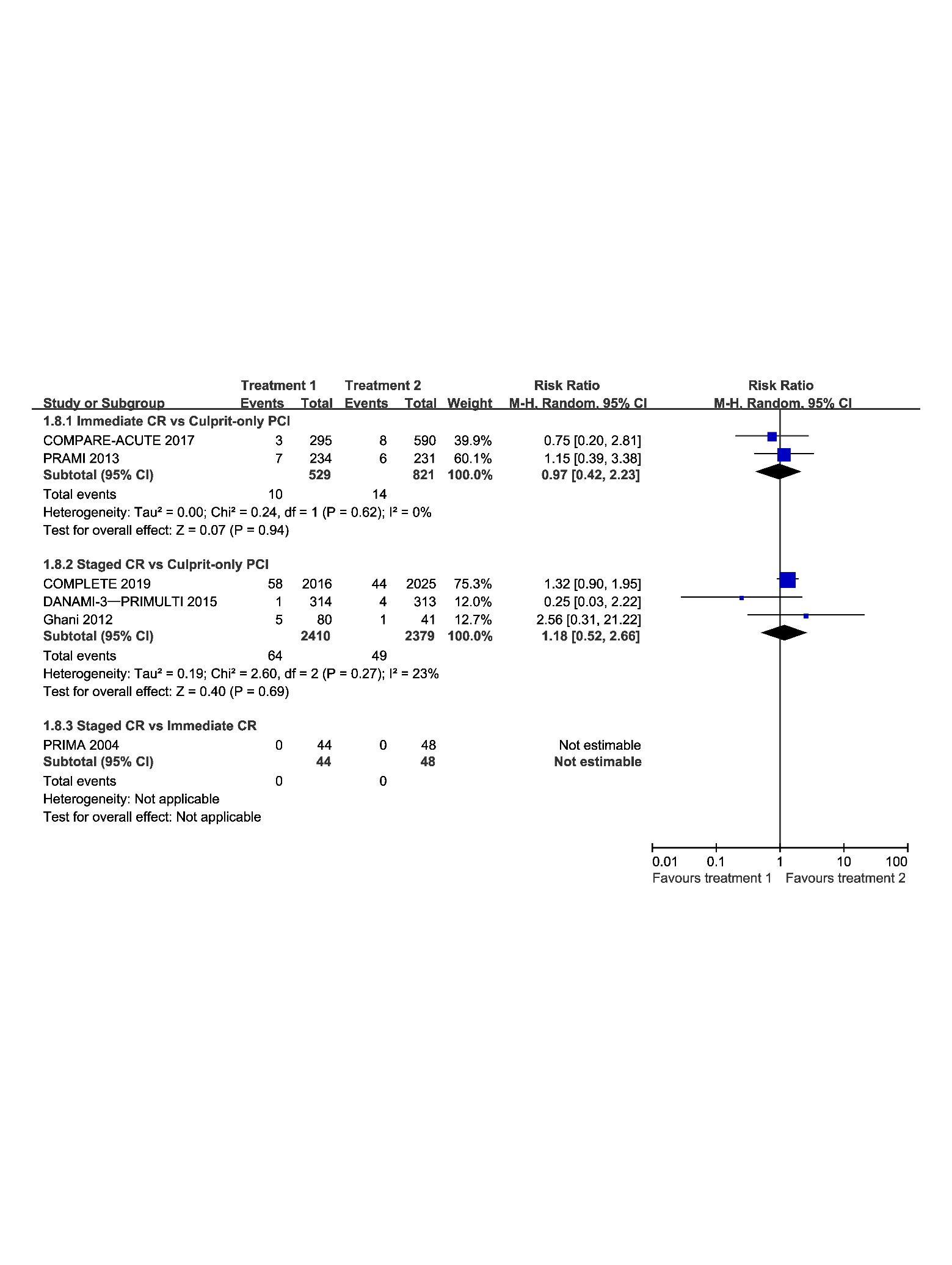
**Figure S9** **Forest Plot of Pairwise Meta-Analysis for Major Bleeding.** CI=confidence interval; CR=complete revascularization; PCI=percutaneous coronary intervention.


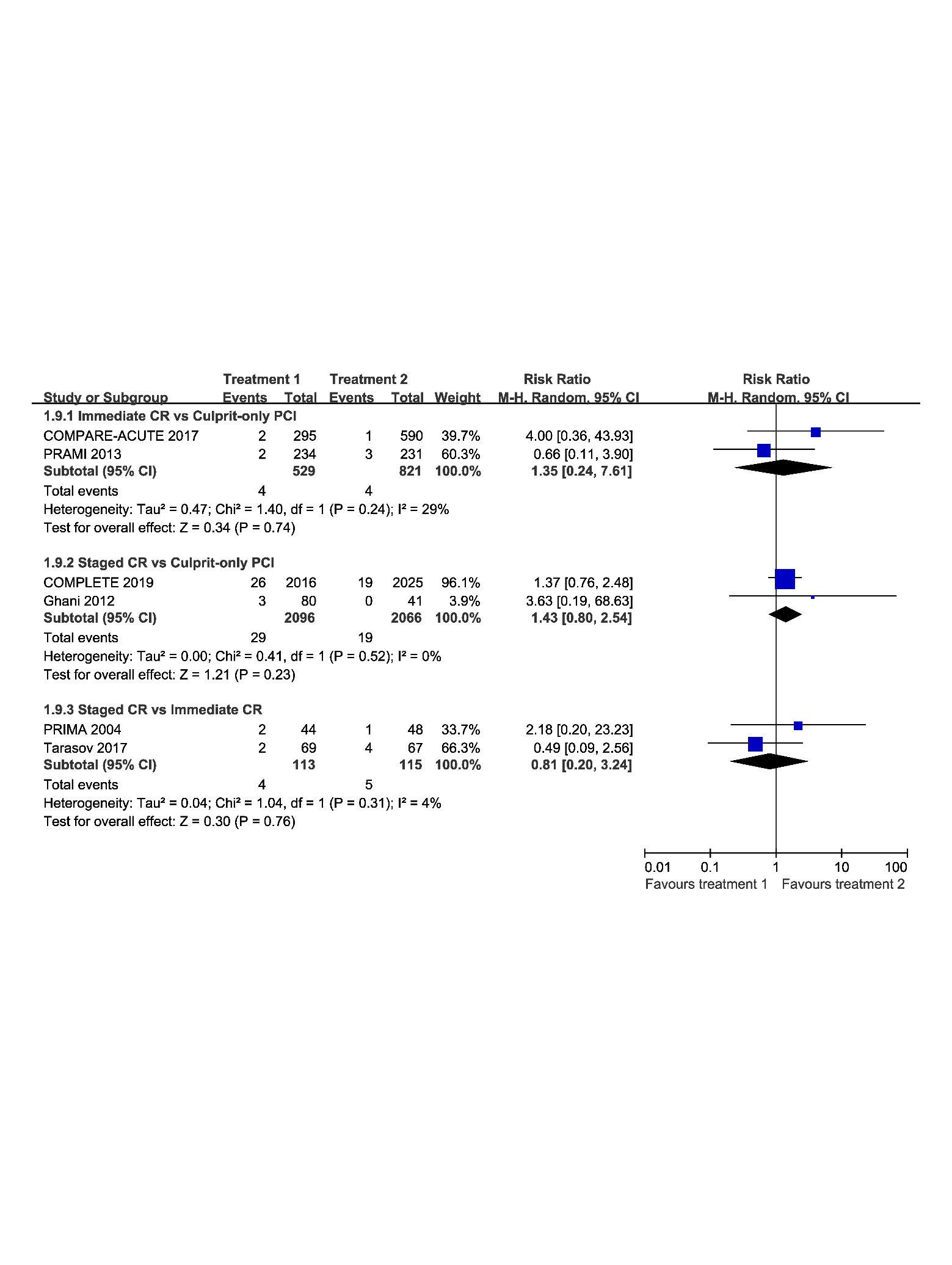
**Figure S10 Forest Plot of Pairwise Meta-Analysis for Stent Thrombosis.** CI=confidence interval; CR=complete revascularization; PCI=percutaneous coronary intervention.

**Figure S11 Rank Probability Analysis Results of All the Efficacy Outcomes.** CR=complete revascularization; PCI=percutaneous coronary intervention.

**
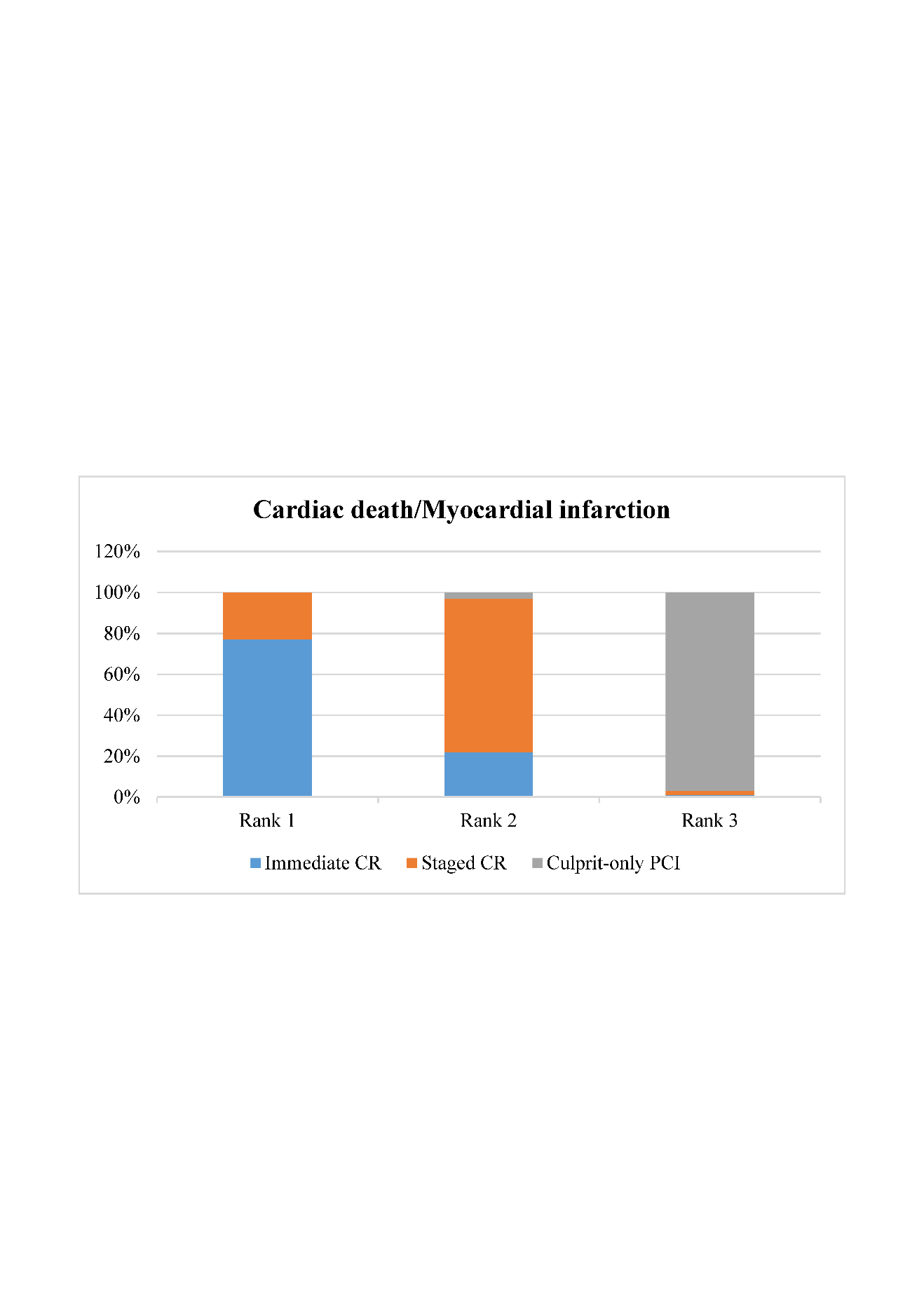
(A) Cardiovascular Mortality/Myocardial infarction**

**
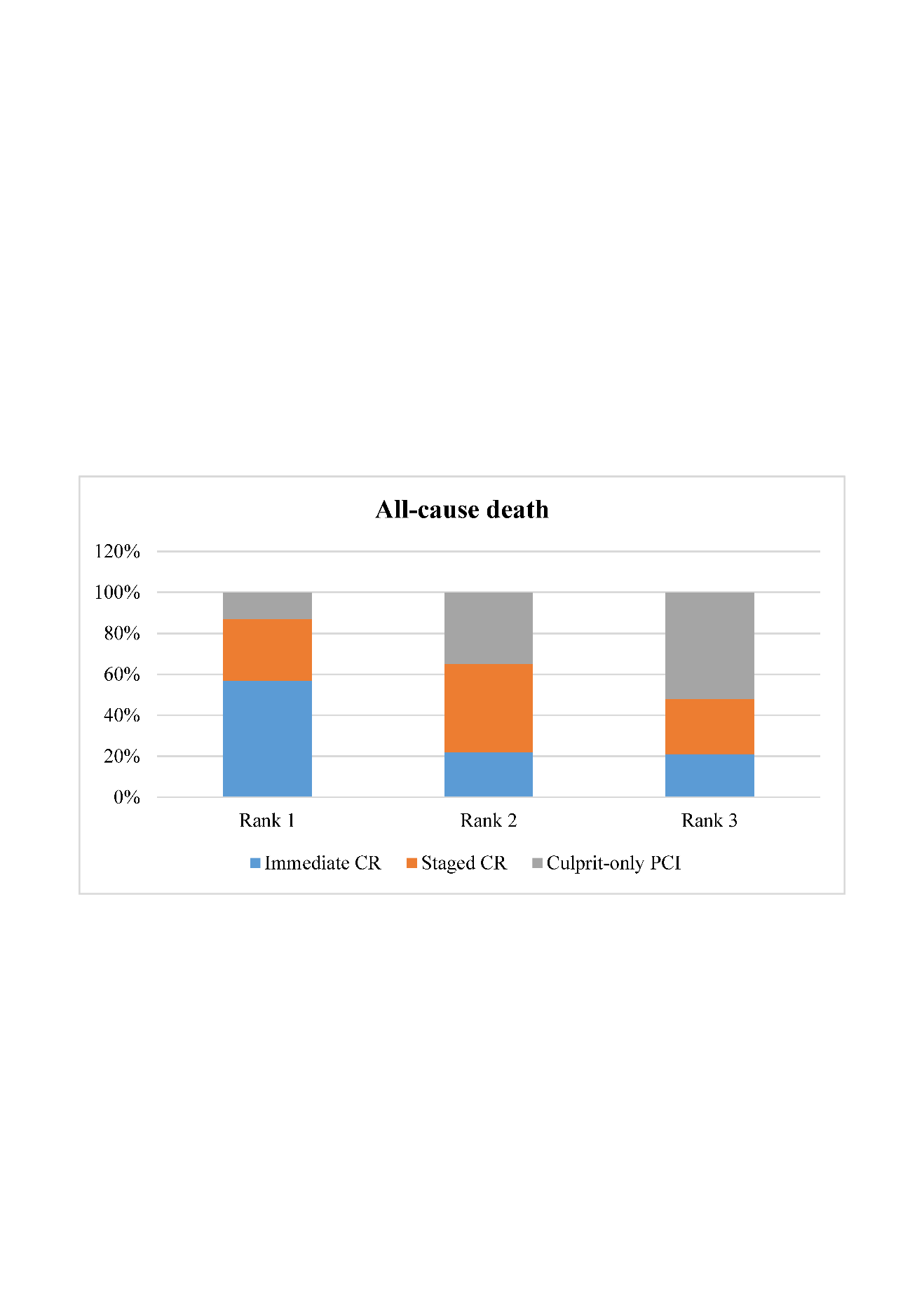
(B) All-Cause Mortality**

**
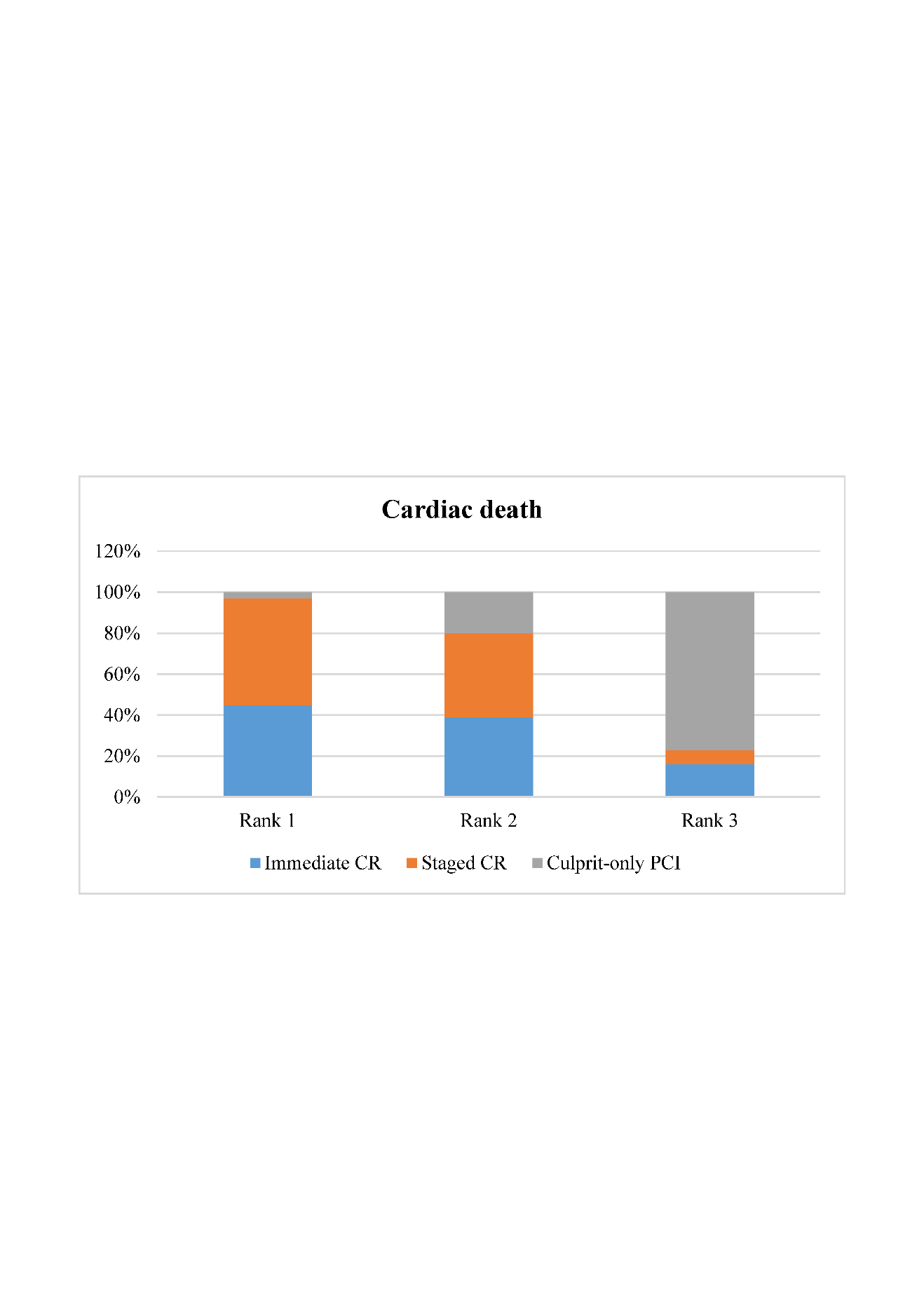
(C) Cardiovascular Mortality**


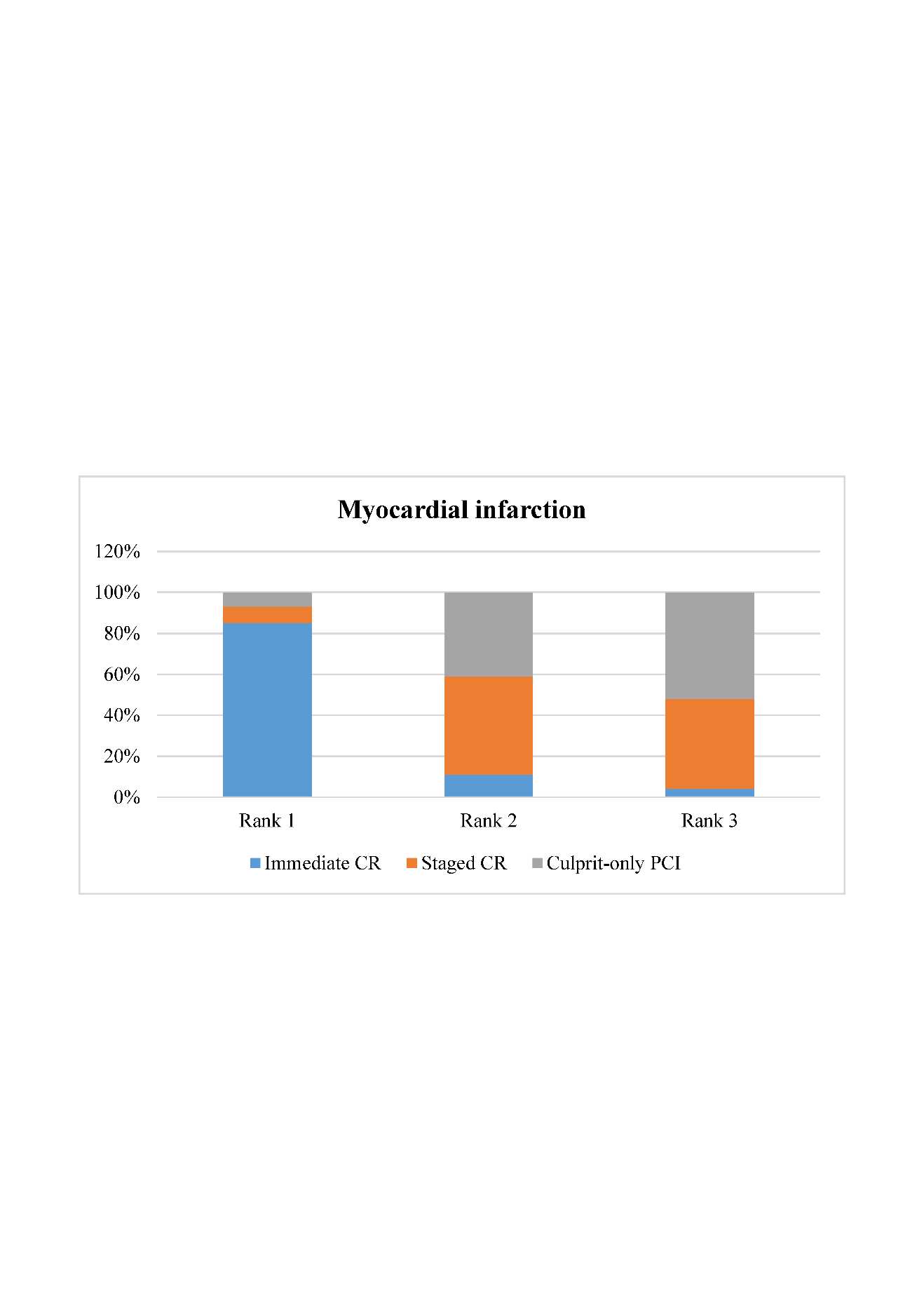
**(D) Myocardial Infarction**

**
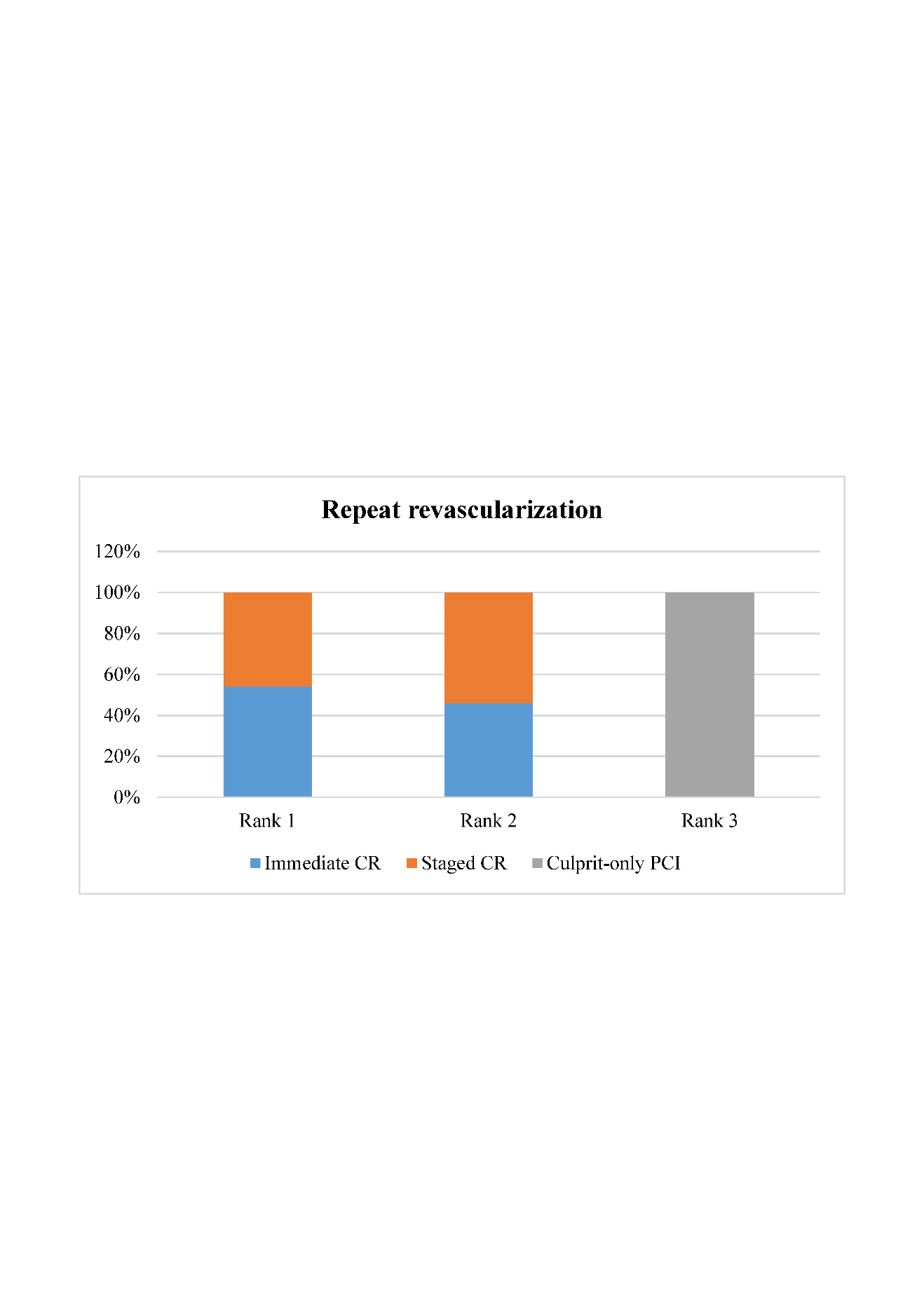
(E) Repeat Revascularization**


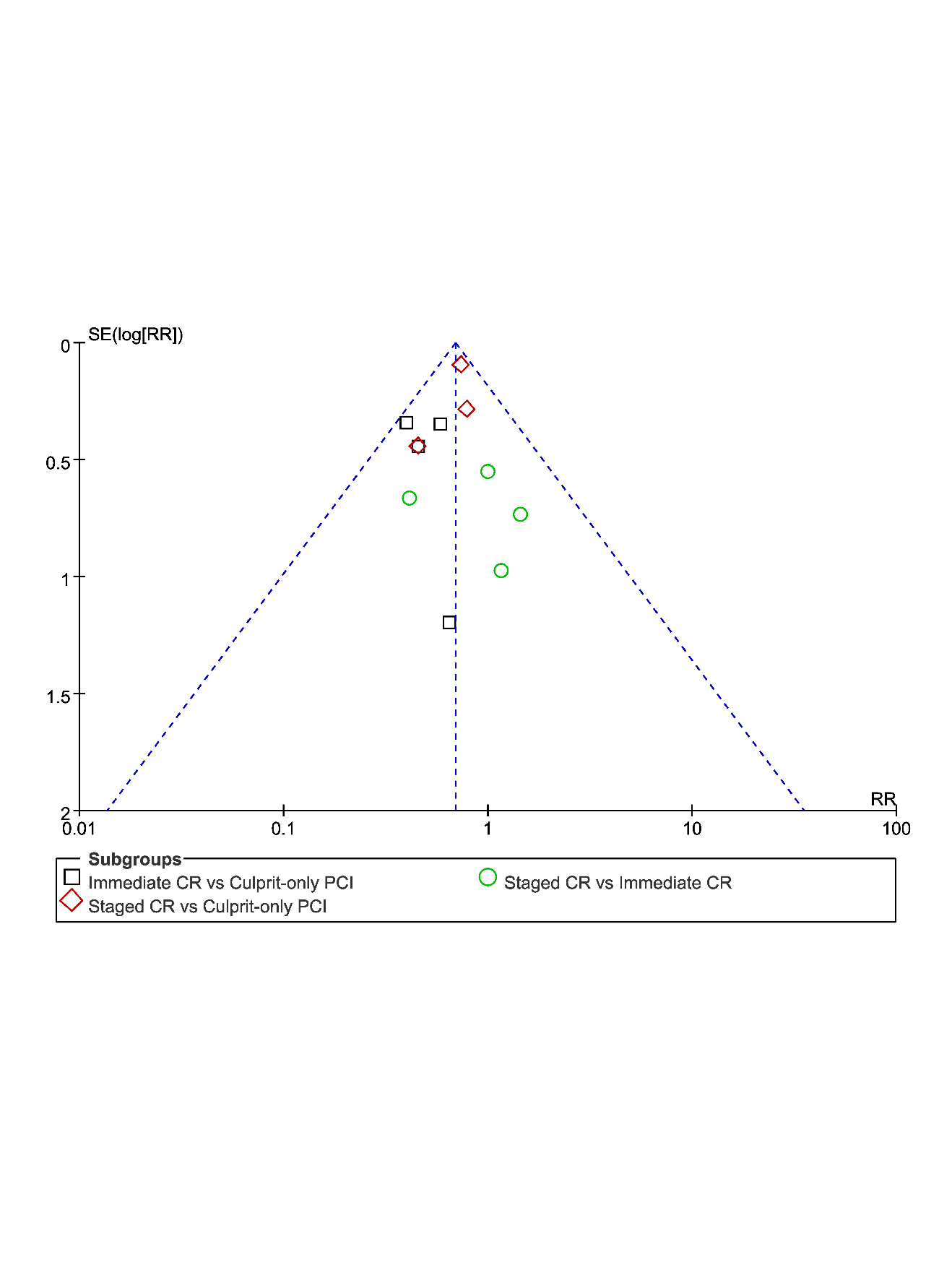
**Figure S12** **Funnel Plot of Studies for the Risk of Cardiovascular Mortality or Myocardial infarction.** CR=complete revascularization; PCI=percutaneous coronary intervention; RR=relative risk.


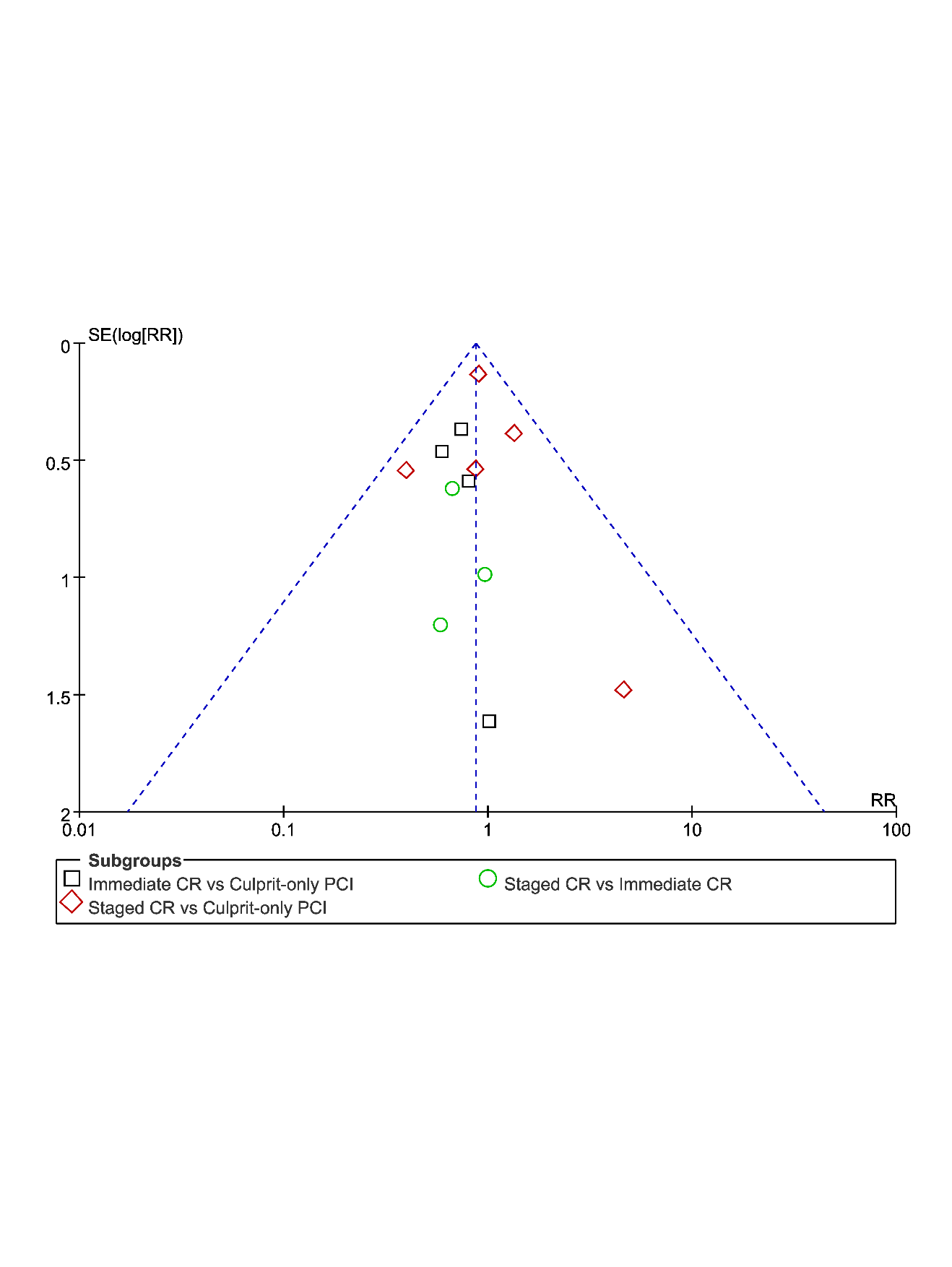
**Figure S13 Funnel Plot of Studies for the Risk of All-Cause Death.** CR=complete revascularization; PCI=percutaneous coronary intervention; RR=relative risk.


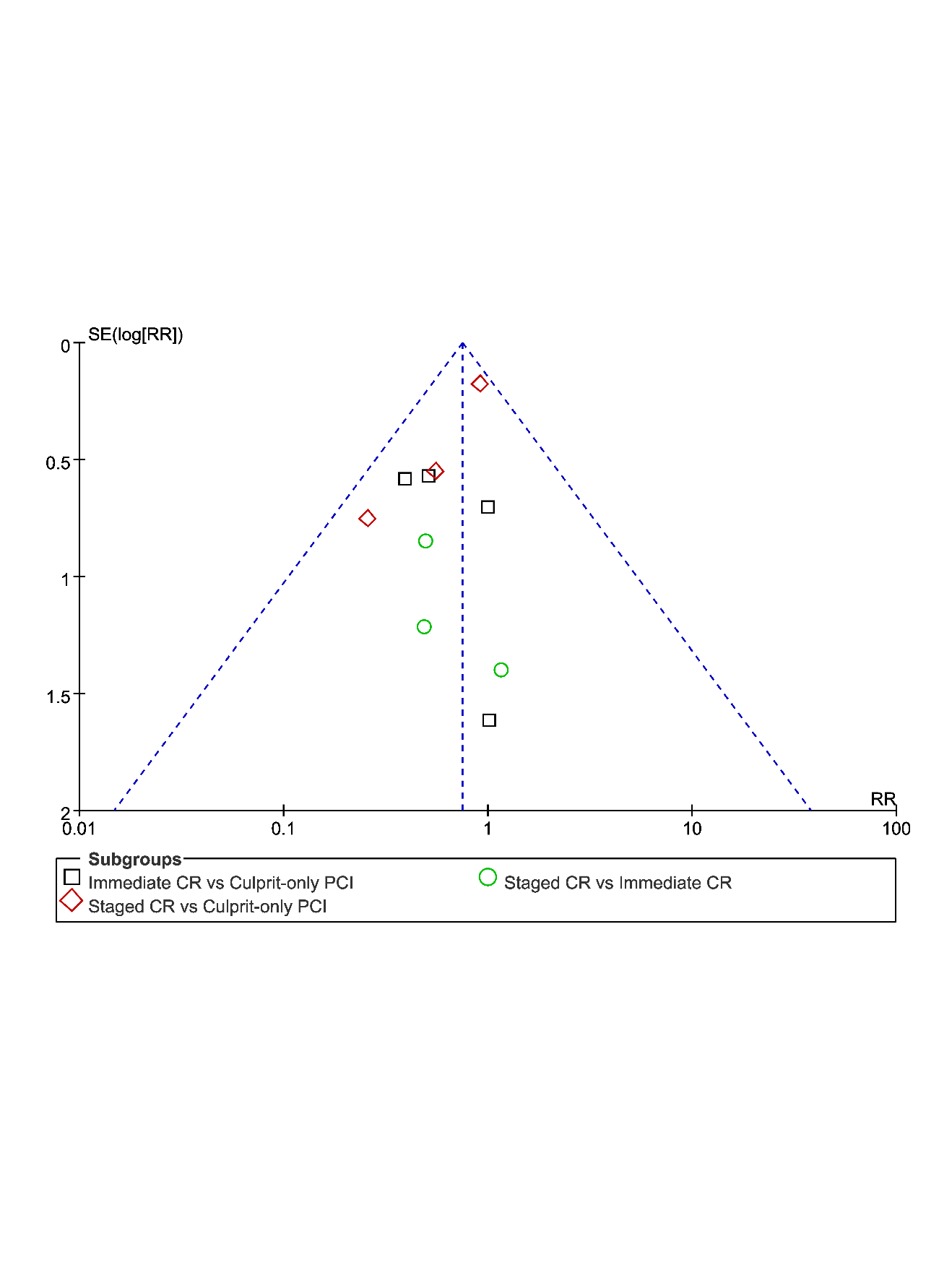
**Figure S14 Funnel Plot of Studies for the Risk of Cardiac Death.** CR=complete revascularization; PCI=percutaneous coronary intervention; RR=relative risk.


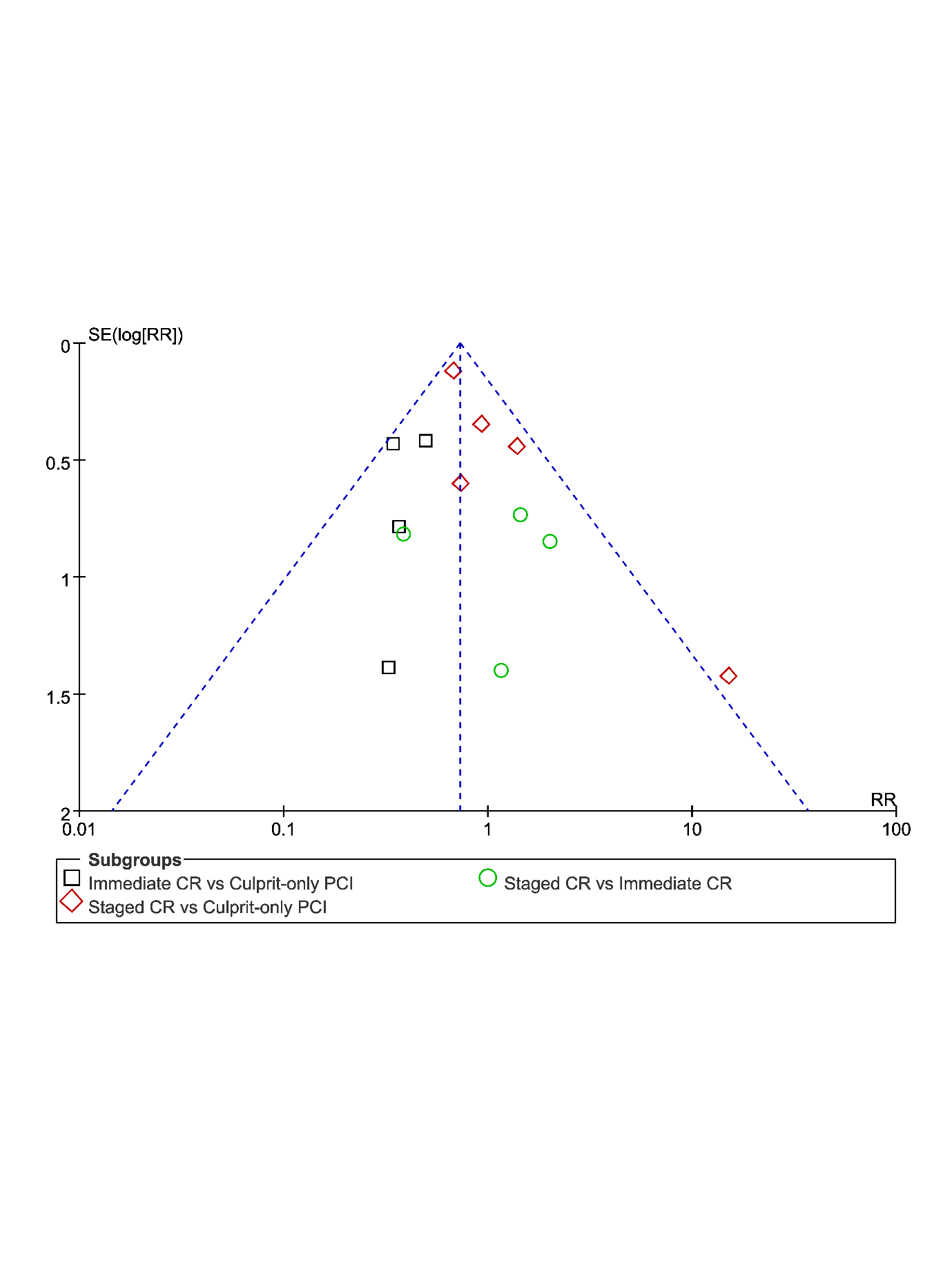
**Figure S15 Funnel Plot of Studies for the Risk of Myocardial Infarction.** CR=complete revascularization; PCI=percutaneous coronary intervention; RR=relative risk.


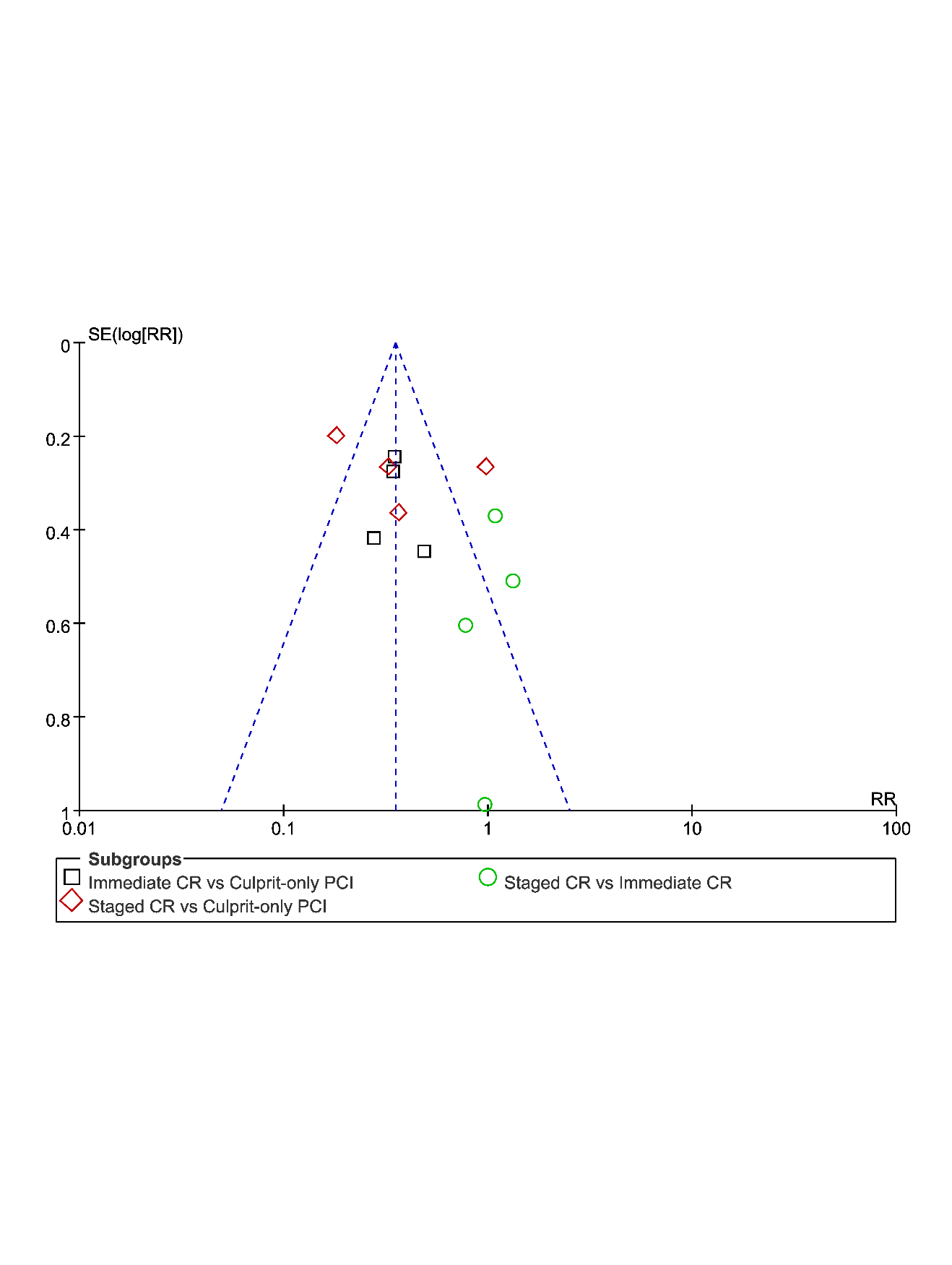
**Figure S16 Funnel Plot of Studies for the Risk of Repeat Revascularization.** CR=complete revascularization; PCI=percutaneous coronary intervention; RR=relative risk.


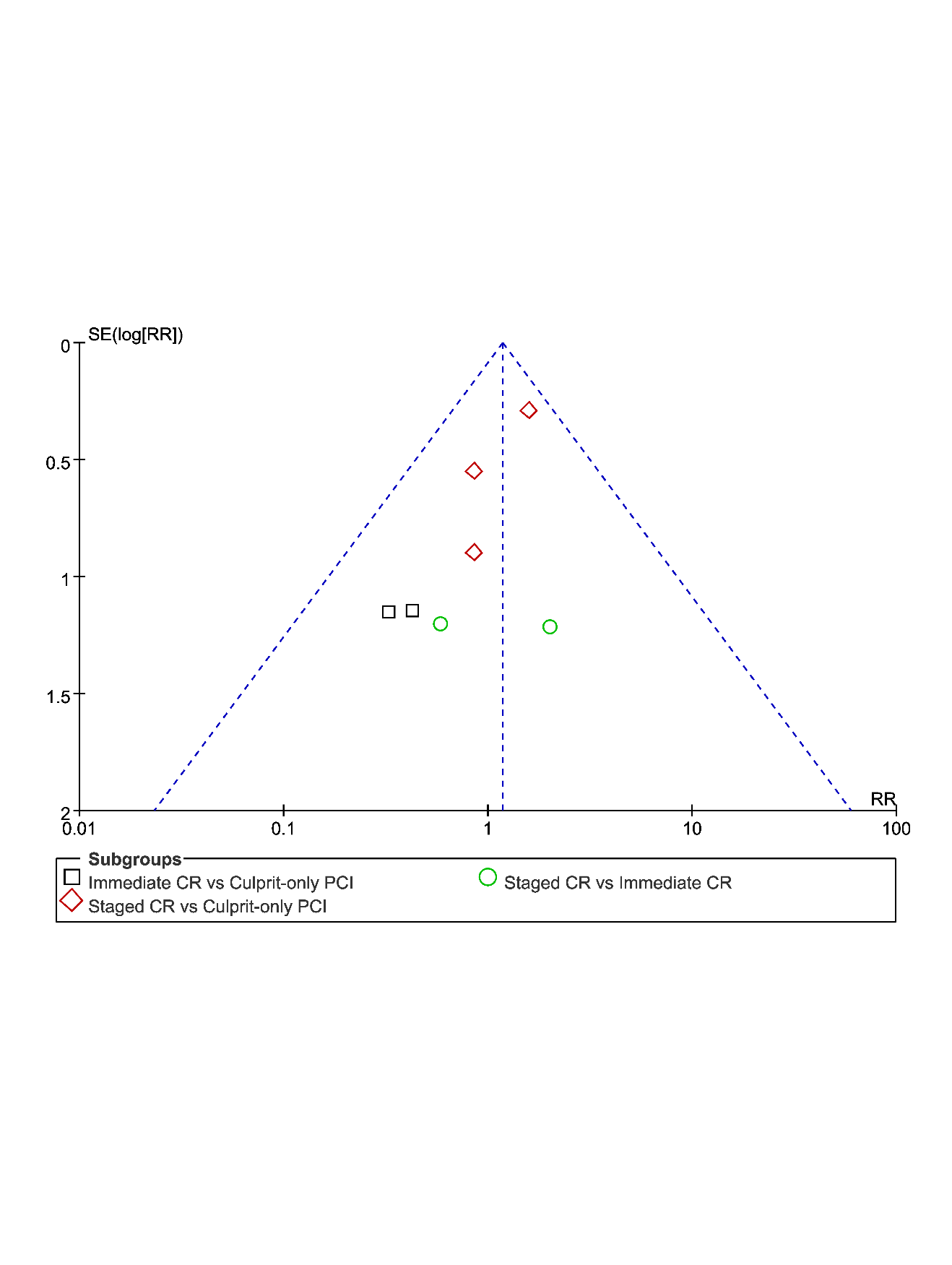
**Figure S17 Funnel Plot of Studies for the Risk of Contrast-Associated Acute Kidney Injury.** CR=complete revascularization; PCI=percutaneous coronary intervention; RR=relative risk.


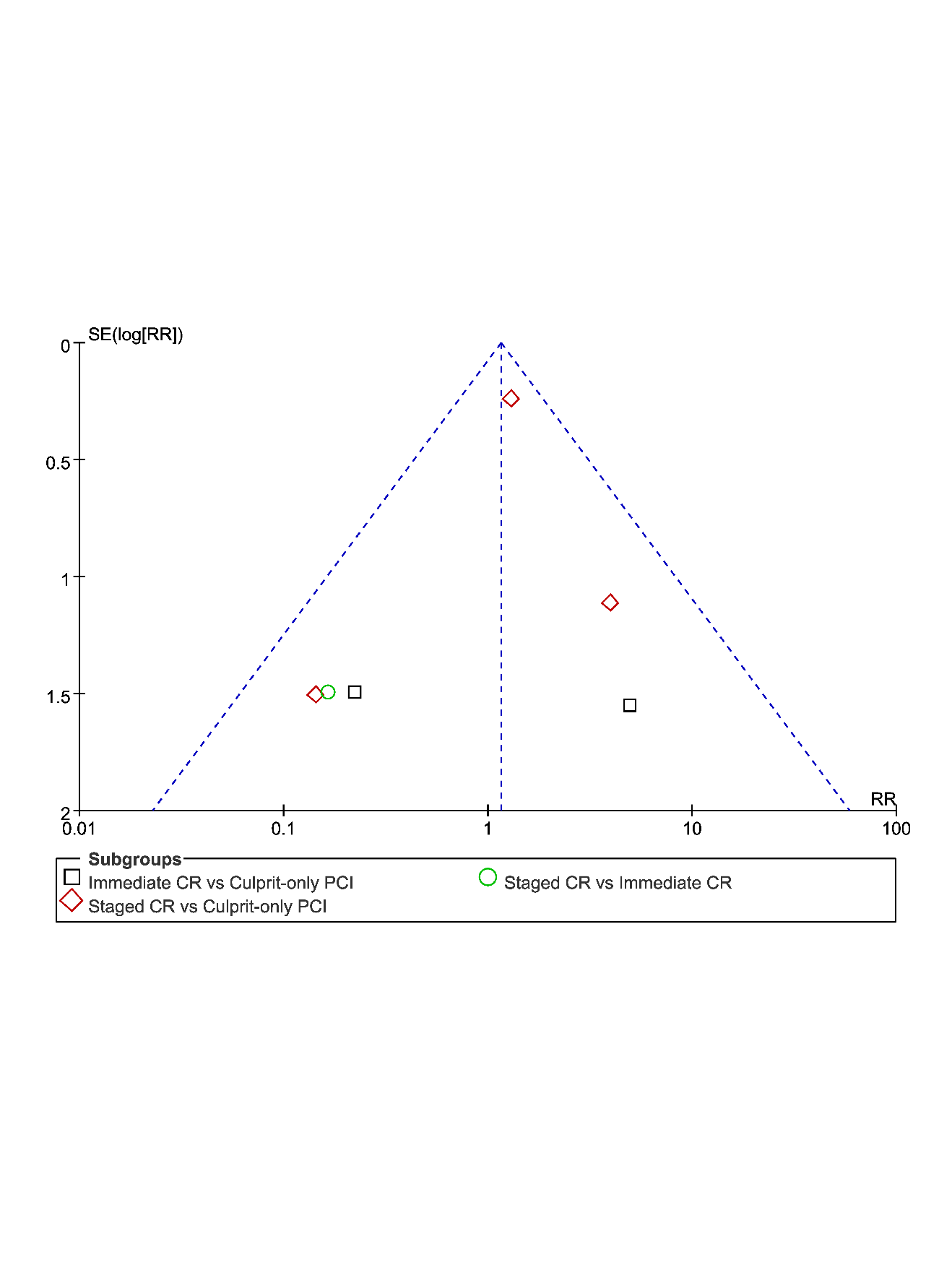
**Figure S18 Funnel Plot of Studies for the Risk of Stroke.** CR=complete revascularization; PCI=percutaneous coronary intervention; RR=relative risk.


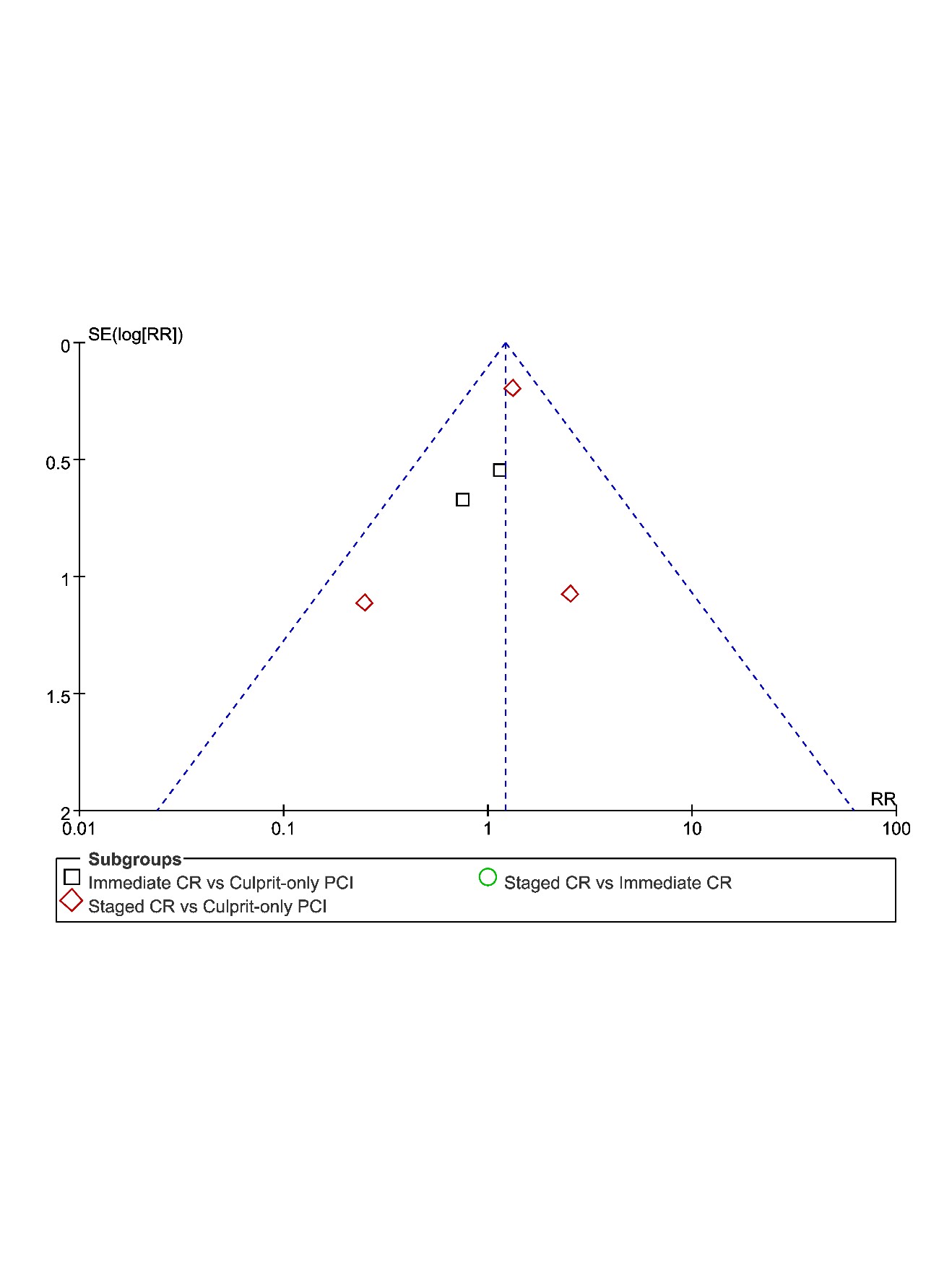
**Figure S19 Funnel Plot of Studies for the Risk of Major Bleeding.** CR=complete revascularization; PCI=percutaneous coronary intervention; RR=relative risk.


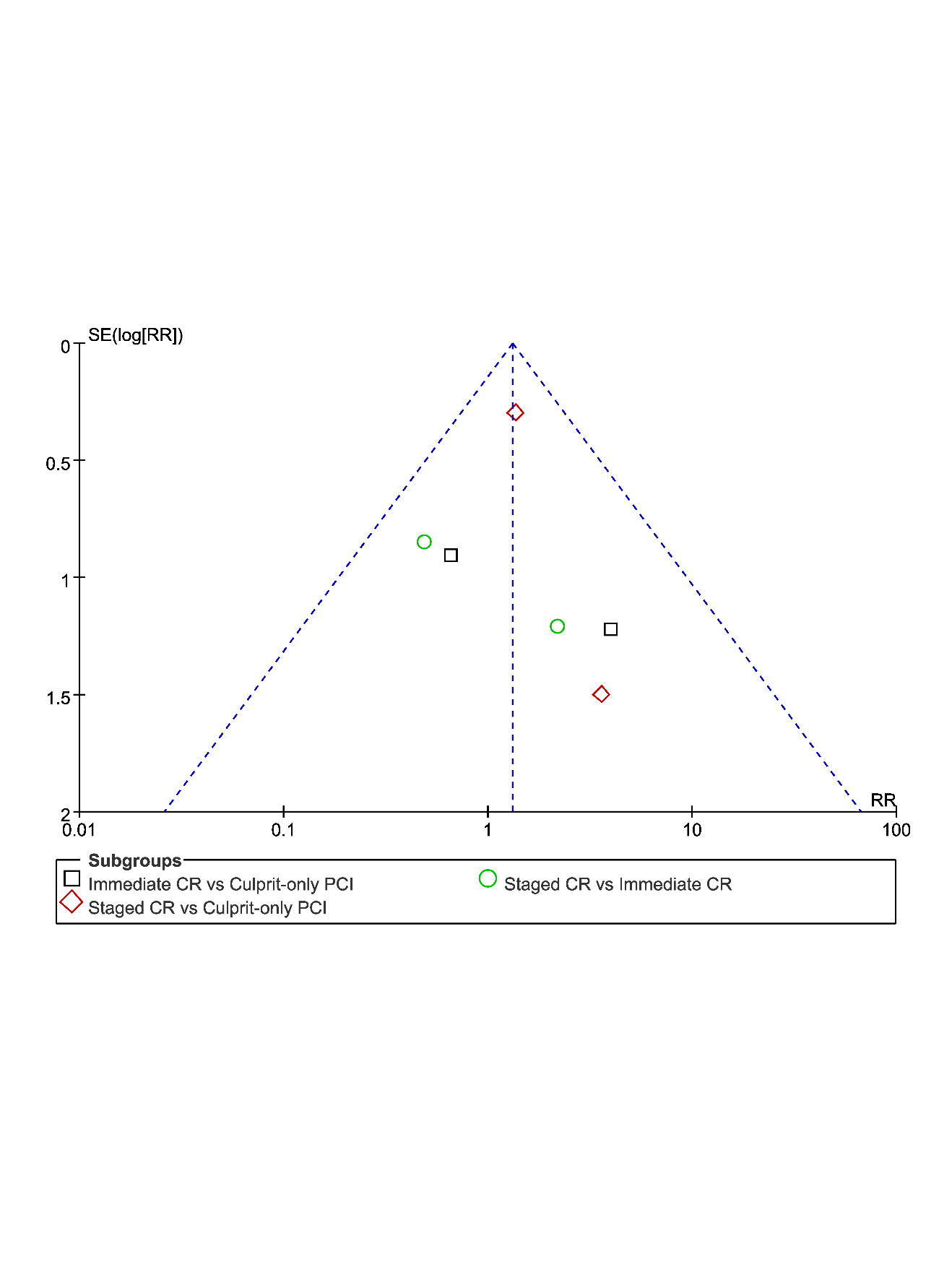
**Figure S20 Funnel Plot of Studies for the Risk of Stent Thrombosis.** CR=complete revascularization; PCI=percutaneous coronary intervention; RR=relative risk.
